# Climate change-driven compound dust storm and heatwave extremes in Central Asia under future CMIP6 scenarios

**DOI:** 10.1371/journal.pone.0359347

**Published:** 2026-11-04

**Authors:** Parya Broomandi, Mehdi Bagheri, Nick Middleton, Ali Mozhdehi Fard, Aram Fathian, Dorna Gholamzade Ledari, Vadim Yapiyev, Ivan Radelyuk, Michael Leuchner, Jong Ryeol Kim

**Affiliations:** 1 Department of Civil and Environmental Engineering, School of Engineering and Digital Sciences, Nazarbayev University, Astana, Kazakhstan; 2 Department of Electrical and Computer Engineering, School of Engineering and Digital Sciences, Nazarbayev University, Astana, Kazakhstan; 3 St Anne’s College, University of Oxford, Oxford, United Kingdom; 4 Water, Sediment, Hazards, and Earth-surface Dynamics (waterSHED) Lab, Department of Geoscience, University of Calgary, Calgary, Alberta, Canada; 5 National Laboratory Astana, Nazarbayev University, Astana, Kazakhstan; 6 Department of Chemistry and Chemical Technology, Toraighyrov University, Pavlodar, Kazakhstan; 7 Physical Geography and Climatology, Department of Geography, RWTH Aachen University, Aachen, Germany; Lanzhou Jiaotong University, CHINA

## Abstract

Central Asia (CA) faces rising temperatures and shifting atmospheric conditions, increasing the urgency to address extreme events like heatwaves and dust storms. This study examines the co-evolution and regional co-variability of heatwaves and atmospheric dust loading, represented by dust extinction aerosol optical thickness (AOT). Using a multi-model ensemble of CMIP6 General Circulation Models (GCMs) and high-resolution reanalysis datasets, we analyzed heatwave indices alongside AOT across six clusters from 1992 to 2100. Results show a substantial rise in heatwave activity, with increases in heatwave frequency and intensity projected across much of CA, particularly under future emission scenarios. AOT trends vary regionally, with both increasing and decreasing trends across clusters, periods, and emission scenarios. Spatial and temporal correlations, evaluated with Benjamini–Hochberg False Discovery Rate (BH-FDR) correction for multiple location-level tests, show regionally heterogeneous dust–heatwave co-variability, with both positive and negative associations across clusters and scenarios. The strongest FDR-supported relationships occur in selected cluster–index combinations, indicating localized and scenario-dependent co-variability rather than a uniform compound dust–heatwave signal across CA. These findings highlight the need for targeted adaptation strategies for CA, which faces intensifying heatwaves and spatially variable dust-related atmospheric conditions. This study supports early warning systems, risk assessments, and mitigation and resilience measures to safeguard vulnerable communities and ecosystems and strengthen resilience to these hazards in line with the Sendai Framework for Disaster Risk Reduction 2015–2030.

## 1. Introduction

CA is highly vulnerable to the impacts of climate change. Its temperature has increased significantly over the past fifty years (approximately 0.30°C per decade), exceeding the global average of 0.19°C per decade [1–5]. Recent assessments confirm that Asia, including CA, has warmed nearly twice as fast as the global average, with 2023–2024 ranking among the warmest years observed [6]. Building on these findings, Fallah et al. 2024 [3] highlight that CA is projected to warm up to 6°C under SSP5–8.5 scenarios by the end of the century.

Compared to any other Asian region, CA suffers from a lack of available data and research on actual and projected climate change impacts [7–11]. Most of the research is centered on examining changes in mean temperature and precipitation [12–14]. Temperature changes across the region exhibit a consistent and clear pattern; however, variations in precipitation are marked by significant differences. Moreover, a limited number of studies exploring the distributions of temperature and precipitation have highlighted the pressing need to focus on extreme weather events, including heatwaves, droughts, and dust storms [15–20].

By examining extreme weather occurrences, previous studies have suggested that there will be a rise in extreme temperatures, the occurrence of droughts, and all heatwave-related indices such as duration, intensity, and frequency [16,20–22]. Excess mortality in the population [23–25] and a reduction in vegetation productivity [26,27] are among the effects of heatwaves on human society and ecosystems. Climate extremes are also interconnected; anthropogenic warming increases the risk of co-occurring and cascading hazards [28], including compound events such as drought and heatwaves [29]. Recent research has emphasized the critical role of compound extreme weather events and their cascading impacts on ecosystems and communities [30]. Similarly, studies such as Adeyeri et al. (2025) [31] highlight the intensification of global heatwaves under the influence of climate change and provide a framework for understanding the multidimensional drivers of extreme heat events [31].

In addition, an extreme heatwave in March 2025 across CA was found to be at least 4°C more intense due to anthropogenic warming, indicating the region’s increased vulnerability [32].

In addition to these extreme temperature-related weather events, a recent analysis of the Taklamakan Desert region found that over 40% of heatwaves coincide with dust storms, indicating a potentially widespread phenomenon in CA [23,33,34].

However, the lack of research investigating the co-occurrence of dust and heatwave phenomena is especially significant, especially in CA, where these risks are expected to increase in the context of climate-change projections. Though variability of dust has been discussed in several studies (ex: Liu et al., 2024 [33]), combined projections that concurrently evaluate the co-evolution of dust and heatwaves across climatic conditions change scenarios are sparse, specifically in CA.

In this sense, the first multi-sector sand and dust storm (SDS) risk assessment within the arid CA is performed by Wang et al. (2024) [35], who found out that more than 18 percent of the territory is at medium-to-high risk of SDS, and hotspots of the concentrations are located near the Aral Sea, southern Xinjiang, and agricultural oases. The atmospheric dust originating in the solonchacks of the Aralkum desert, as well as the bare toxic bottom of the Aral Sea, can be transported over thousands of kilometers, influencing atmospheric conditions far beyond the region. As an extension of this knowledge, the present study extends previous research by examining the joint evolution of dust and heatwave in CA under future emission scenarios [35–37].

Heatwaves and dust storms co-exist under the influence of physical interactions. Prolonged heatwaves enhance surface drying, reduce soil moisture and vegetation cover, making the soil more susceptible to aeolian erosion. Dust particles then alter the surface energy balance – the absorption and scattering of solar and infrared radiation – which can enhance near-surface heating and prolong heatwave conditions [38,39].

Rising temperatures can further intensify this interaction by reducing relative humidity, increasing evaporation, and enhancing drought frequency, which together promote soil desiccation and dust emission [40–42]. There are clear indications of an increase in the frequency and concentration of dust storms in parts of the Middle East, North Africa, and the eastern Mediterranean [43–48]. Their significant impacts extend beyond human health and air quality to include agriculture, the environment, power generation, industry, transportation, and water quality [49]. In addition, a number of researchers have shown a correlation between dust periods and heatwaves. Wang et al. (2009) found a strong positive relationship between daily air temperature variations and the occurrence of dust storms in Inner Mongolia, China [50]. Limani et al. (2006) found that fine particle concentrations in southeastern Spain were higher during heatwaves than in subsequent periods [51]. The current cases indicate the mutual enhancement of local meteorological and terrestrial conditions, which contribute to the development of dust events and heatwaves.

Despite these findings, the mechanisms and future evolution of dust–heatwave interactions under climate change remain insufficiently understood, particularly in CA. This lack of studies has created serious gaps in knowledge about the co-occurrence, causal pathways as well as the consequential effects in this susceptible area. Recent studies have addressed climate extremes and their risks in CA, such as the expected increase in the intensity of heatwaves [23], dust variability and subsequent exposure to the population, and the overall the large-scale climate change trend [3]; however, their future co-evolution under climate change scenarios remains largely unexplored. It is this gap in knowledge that gives motivation to current study.

The predicted combination of dust storms and heatwaves is likely to make CA face tremendous challenges, and its consequences are likely to affect not only human health but also the environment and economic conditions. High concentration of particulate matter on prolonged heatwaves has been recorded to increase respiratory and cardiovascular morbidity as noted in arid areas in East Asia and the Middle East [52]. Due to insufficient understanding of the impacts of climate change, there are significant economic, environmental, and social risks in the region [53]. Addressing these risks requires robust and adaptive climate management strategies.

While previous studies have examined historical combined events such as heatwave-dust storm co-occurrences (e.g., Liu et al., 2024 [33]), this study is the first to project the future co-evolution of these combined hazards in CA under climate change. To address this gap, we develop an integrated modelling framework that combines bias-corrected CMIP6 projections, regional clustering, and machine-learning techniques to analyse the spatiotemporal interactions between atmospheric dust (AOT) and heatwave indices.

The objectives of this study are: (1) to examine spatiotemporal variations of heatwaves across CA and identify regions most vulnerable to extreme heat; (2) to analyze spatial and temporal patterns of dust events and assess their environmental and societal implications; and (3) to assess the correlation between heatwave-related indices and dust events from 1992 to 2100, examining their combined effects to enhance the development of integrated early warning systems.

By addressing these objectives, this study provides important insights into the nexus of heatwaves and dust events, with the aim of supporting climate adaptation strategies and resilience efforts tailored to CA’s unique vulnerabilities.

## 2. Materials and methods

### 2.1. Study domain

The Central Asian Plains extend from the western shores of the Caspian Sea to the foothills of the Altai, Tian-Shan, and Pamir Mountains in the east (Fig 1A). CA includes the countries of Uzbekistan, Kazakhstan, Kyrgyzstan, Tajikistan, and Turkmenistan [22,54]. For spatial analysis, the region was divided into provinces, which are listed in S1 Table along with their corresponding numerical codes. These provincial codes are also depicted in Fig 1B and provide a geographic context for clustering and subsequent analysis.

**Fig 1:**
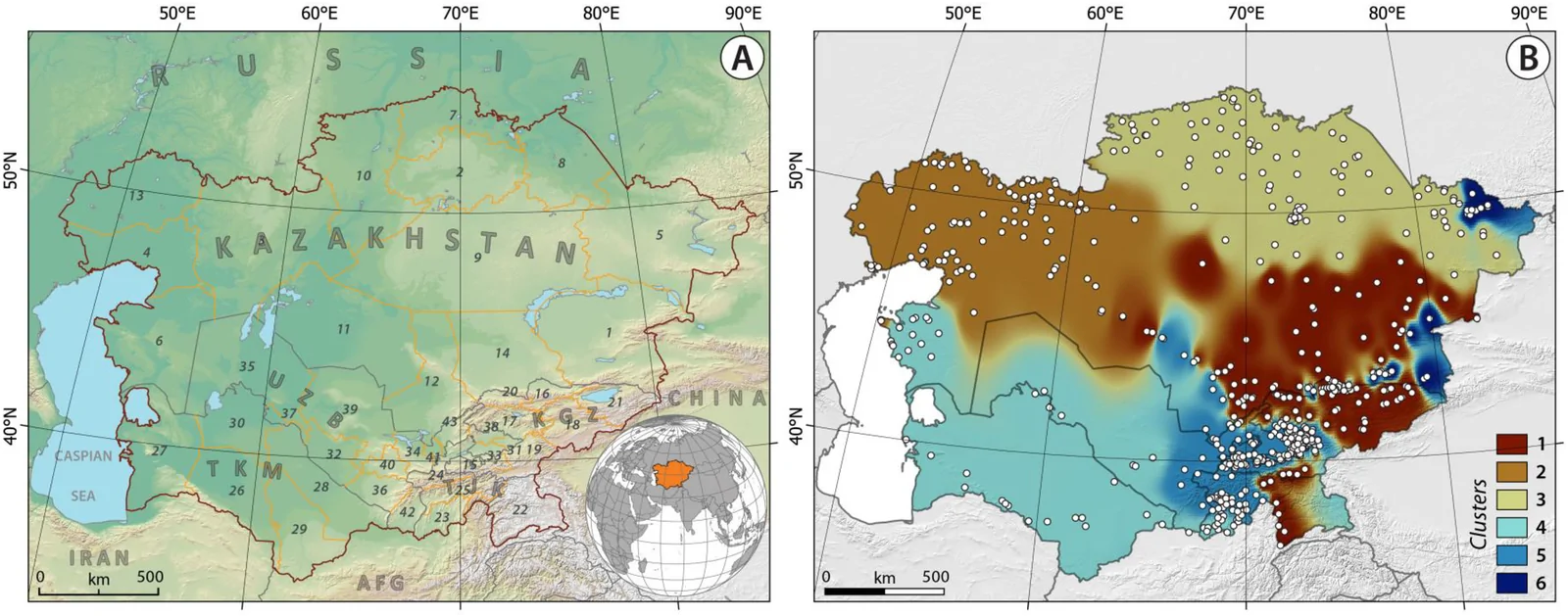
(A) The area of interest, CA consists of Kazakhstan, Kyrgyzstan, Tajikistan, Turkmenistan, and Uzbekistan. The numbers refer to provinces listed in S1 Table; **(B)** Cluster zoning of CA based on the maximum temperature, AOT, and geographical characteristics including latitude and longitude. Please note that the white dots represent the locations included in our database, while the color scale corresponds to the six identified clusters in CA.

CA is geographically divided into two distinct climatic regions, northern and southern, based on differences in synoptic processes and meteorological conditions [55,56]. CA has extensive drylands and rangelands transitioning from steppe in the north to semi-desert landscapes in the south. CA primarily experiences a dry to semi-arid climate, influenced mainly by mid-latitude westerly winds. The northern part has a cold continental climate, but the southern region is characterized by hotter and drier conditions. Significant temperature variations, limited precipitation, and high evaporation rates are widespread throughout most of CA. The average annual temperature in the northern region ranges from 2 to 9 °C, while in the southern region it rises to 6–11°C [56]. The annual precipitation across the region varies from approximately 80 mm in arid lowland areas to around 200 mm in steppe and foothill zones, while mountainous regions receive considerably higher precipitation due to elevation and local climatic conditions. In the desert areas of the western Balkash shore, Kyzylkum, Karakum Deserts, and Betpak-Dala, precipitation is typically below 100 mm [54,57]. In CA, the warm season is from May to September [58].

### 2.2. Data

The present research obtained daily meteorological data from the ERA5 (ECMWF Reanalysis v5) reanalysis database, encompassing average daily 2-m air temperature (°C) (maximum and minimum), precipitation (mm/day), and 10-m wind speed (m/sec) [59]. The study used monthly mean dust extinction aerosol optical thickness (AOT) data at 550 nm from the MERRA-2 reanalysis with a spatial resolution of 0.5° × 0.625°. These datasets were used to assess meteorological drivers and dust activity across CA. The dataset covered the period 1992–2021 and included 700 locations across CA. In this study, each “location” corresponds to a predefined geographic coordinate for which meteorological data were extracted from the nearest ERA5 grid point. These locations were selected using available geographic datasets and city-level information across the five Central Asian countries: Kazakhstan, Kyrgyzstan, Tajikistan, Turkmenistan, and Uzbekistan. The selected locations include urban, suburban, and rural areas to ensure broad spatial coverage across the region. The World Cities Database and World Population Review were used to obtain the spatial distribution and geographic coordinates of populated areas. S1 Table lists all the provinces in CA and the numerical identifiers and country codes.

The Copernicus Climate Change Service (C3S) provided GCMs of different spatial resolutions of the Copernicus Climate Change Service phase 6 (CMIP6) in order to determine the impact of climate change on key variables. S2 Table outlines the CMIP6 models that were used in the present investigation, including their spatial resolutions and sources. Ten models were identified and chosen as a mutually consistent group that could be consistently used to provide all the necessary variables (maximum temperature, minimum temperature, precipitation, and wind speed) both in the past and in the future, thus guaranteeing homogeneity in the methodology of all analyses [60].

The scenario ensemble was selected to include SSP2–4.5 and SSP5–8.5 because these pathways represent two contrasting climate futures: a moderate stabilization pathway and a high-emission pathway, respectively. This selection allows assessment of moderate and severe climate outcomes associated with combined heat–dust hazards in CA. Historical CMIP6 projections were used for 1992–2014, consistent with the end year of CMIP6 historical simulations. Future analyses were conducted for two 30-year periods: the near future (2022–2051) and the far future (2071–2100). The intermediate period of 2015–2021 was not included in the future analysis because it falls between the end of the CMIP6 historical simulations and the defined near-future assessment window.

In this research, spatial interpolation at 0.1° (0.1° × 0.1°) was only applied for visualization purposes using the inverse distance weighting (IDW) method to create more flattened spatial patterns. This method was used due to the fact that it provides a simple and reliable way of representing the spatial gradients and keeping the general structure of the original data. It could be mentioned that all analytical operations were performed on datasets in their native spatial resolutions: ERA5 at 0.25 and CMIP6 native resolution of each model, which is different among models. In addition, in the analysis it was subdivided into urban, suburban, and rural population centers (i.e., cities and towns) to better assess population exposure and associated vulnerabilities, as opposed to using a uniform grid and considering the whole of CA.

AOT was divided into four discrete categories using intensity threshold to measure the levels of dust in the atmosphere. The low-intensity group includes the AOT values located in the lower quartile (0–25^th^ percentile), which means that there is a low level of dust and quite clean conditions. The medium-intensity category comprises of the AOT values between 25^th^ and 75^th^ percentiles which represent normal dust conditions. The high-intensity category is between the 75^th^ and 90^th^ percentile which indicates above normal dust levels and increased activity of dust. Lastly, the very-high-intensity category includes AOT values above the 90^th^ percentile that denotes high dust activity and the worst dust events. The use of these percentile cutoffs (25^th^, 75^th^, 90^th^) is consistent with the current practices in climate and aerosol research, where a percentile-based classification is successful in making the distinction between normal and extreme events; the 90^th^ percentile is commonly used to identify extreme aerosol events [61].

A detailed description of the K-means clustering procedure and the calculation of heatwave-related climate indices is provided in the Supplementary Material (Sections S1 and S2, respectively). For the heatwave analysis, the Tx90 threshold was calculated separately for each 30-year study period and location. Consequently, the resulting heatwave indices characterize heat extremes relative to the contemporaneous temperature distribution of each study period rather than exceeding a fixed historical Tx90 threshold. Thus, projected changes in HWN, HWD, HWF, HWA, and HWM should be interpreted as changes in the frequency, duration, and intensity of relative heat extremes within the corresponding climatological period. Temporal trends in the heatwave indices were quantified using Sen’s slope, which was used to estimate the magnitude and direction of change in HWN, HWD, HWF, HWA, and HWM. The clustering was performed using temperature, dust, and geographic variables. The optimal number of clusters was selected using the elbow method based on the within-cluster sum of squares (WCSS), with silhouette scores used as an additional diagnostic criterion. The WCSS curve showed a sharp decrease at lower k values and a clear reduction in marginal improvement after k = 6. In addition, the silhouette score for k = 6 was higher than those obtained for the adjacent clustering solutions, k = 5 and k = 7. Therefore, the six-cluster solution was retained because it provided a balanced and interpretable regionalization scheme without unnecessary over-partitioning. Sensitivity results for alternative clustering solutions are provided in Supplementary Section S1.

### 2.3. Bias-corrected climate modelling and scenario analysis

Future climate projections for CA were derived from multiple GCMs using two climate scenarios from the CMIP6 archive: SSP2–4.5 and SSP5–8.5. These scenarios address medium and high global challenges for adaptation and mitigation, respectively, and reflect the expected global effective radiative forcing up to 2100 [62].

To ensure robust estimates, a hybrid semiparametric bias correction method [63] was applied to adjust daily values of temperature, precipitation, and wind speed against historical ERA5 observations, aligning the statistical distributions of the modeled data with the observations. This method corrects systematic differences between GCM outputs and reference data by adjusting the modeled distribution to match the statistical characteristics of historical observations. It was applied separately to each GCM and to each meteorological variable before ensemble averaging. The approach is suitable for this study because it adjusts both mean conditions and distributional characteristics relevant to climate extremes, including percentile-based thresholds used for heatwave-index calculations [60]. Site-specific transfer functions were extracted from the historical overlap period (1992–2014) and applied to future projections (2022–2100). This bias correction method adjusts for systematic biases while preserving the long-term climate change trends and sensitivities inherent in the GCM outputs, ensuring that the predicted signals remain intact. After bias correction, group means were generated using an equal weighting approach (“one model, one vote”), in which each bias-corrected GCM contributed equally to the final ensemble means, reducing the uncertainties inherent in individual model predictions [60]. Bias-correction performance was evaluated using daily paired values from the GCM ensemble and ERA5 at the evaluated locations during the historical evaluation period, with the resulting error statistics averaged over the study domain. Further methodological details on bias correction techniques, statistical tuning procedures, and model performance evaluation are available in the Supplementary Materials (Sections S3 and S4).

#### Feature selection based on random forest analysis.

The objective is to leverage a random forest (RF) algorithm for feature selection within a Long Short-Term Memory (LSTM) network model to improve the prediction of variations between AOT and key meteorological parameters [63–65]. The relative importance of variables associated with AOT in CA was evaluated through a Feature Importance (FI) analysis using RF. Precipitation, 10-meter wind speed, and maximum and minimum 2-meter temperatures were subjected to FI analysis. Only these meteorological variables were used as predictors, with AOT as the target variable. The RF model was also used to determine key predictors and prioritize predictors of dust extinction AOT whose importance scores fell between 1 (the most significant one) and 0 (the least important one). This analysis was not done to develop a predictive model, but only to evaluate relative feature importance. To ensure that the results were reproducible and that the evaluation framework was the same across all clusters, a fixed random seed (random_state = 12) was chosen to perform a 75/25 division of data into training and test sets; however, the goal to rank predictors rather than to optimize predictive accuracy.

In this regard, cluster-specific RF regression was implemented using the scikit-learn RandomForestRegressor. RF models were built individually for each meteorological cluster to estimate AOT using the covariates of Tmax, Tmin, wind speed and precipitation. Because the RF analysis was used primarily to estimate the relative feature importance of the meteorological predictors rather than as the final predictive model, no formal grid search, random search, or Bayesian hyperparameter optimization was performed. A fixed RF configuration was used, with n_estimators = 100, max_depth = None, min_samples_split = 2, min_samples_leaf = 1, max_features = 1.0, bootstrap = True, criterion = “squared_error”, and random_state = 12. The mean decrease in impurity (MDI) criterion was used to derive feature importance and measure the contribution of each predictor to the impurity reduction across all trees in a cluster. Although MDI is widely used in RF models, it can be influenced by predictor characteristics and correlations among variables. Therefore, the reported FI values should be interpreted as relative indicators of predictive importance rather than definitive measures of causal influence or absolute predictor dominance. The generated importance scores were plotted to highlight the highest-ranking predictors. The analysis showed that Tmin, Tmax, wind speed, and precipitation were consistently important statistical predictors of AOT in all climatic clusters.

#### LSTM.

An LSTM method is implemented to predict AOT in CA, leveraging meteorological parameters. LSTM is a recurrent neural-network architecture designed to capture temporal dependencies in sequential data [66,67]. Artificial neural networks identify relationships within data by adjusting the connection weights between neurons [68,69]. This capability is especially beneficial when working with large datasets, as it allows for the essential task of uncovering underlying dependencies based on historical observations, as illustrated in S2 Fig [66,70].

In this study, the LSTM-based future AOT estimates are interpreted as conditional statistical projections of atmospheric dust loading rather than explicit dust-emission simulations. The model was trained to learn historical relationships between meteorological predictors and MERRA-2 dust extinction AOT. Applying this trained relationship to bias-corrected CMIP6 meteorological projections assumes that the historical meteorology–AOT relationship remains sufficiently stable over the future projection periods. This stationarity assumption is necessary because future dust extinction AOT was not directly obtained from CMIP6 aerosol outputs. Therefore, the projections reflect AOT changes associated with future temperature, precipitation, and wind-speed conditions, but they do not explicitly represent future changes in dust-source extent, land cover, soil properties, vegetation dynamics, or anthropogenic aerosol-emission inventories.

The model was trained on historical ERA5 meteorological data and MERRA-2 AOT values using a 70/30 training/validation split, and temporal separation was ensured to prevent data leakage and account for autocorrelation. To further evaluate temporal transferability, the LSTM was also tested using a late-historical holdout period, in which earlier historical years were used for training and the most recent historical years were retained for independent validation. Each input sequence consisted of a 10-month rolling window of meteorological variables (wind speed, precipitation, maximum and minimum temperatures) that were used to predict the next month’s AOT. Hyperparameters, including the number of LSTM units (from 32 to 512), the number of stacked LSTM layers, and the choice of optimizer (Adam or RMSprop), were tuned using 300 trials with 2 runs per trial to minimize validation loss (val_loss). Validation loss (val_loss) was used as the main selection criterion during hyperparameter tuning.

Overfitting was reduced by applying early stopping (wait = 200 epochs), regularization of the dropout regularization (rate = 0.2) between layers, and normalization of all inputs to the range [0,1]. The patience value of 200 epochs was intentionally used to avoid premature stopping caused by short-term fluctuations in validation loss, and the early-stopping callback was implemented with restoration of the best validation-loss weights. Therefore, the final model retained the weights from the epoch with the lowest validation loss rather than the final training epoch. The final LSTM architecture consisted of four stacked LSTM layers with 128, 160, 160, and 32 units, respectively, followed by a dense output layer, and was compiled using the RMSprop optimizer and trained to minimize the mean square error (MSE). Models were all trained using TensorFlow (Python) with fixed random seeds to ensure reproducibility.

The LSTM architecture is also described in detail with the mathematical formulation of all the gates and statistical tests in the Supplementary Material (Section S5). It is necessary to note that in calculating the AOT trends only the data during the warm season of all the studied locations and timeframes were filtered to better capture dust-prone conditions. Each AOT time series was decomposed using seasonal trend decomposition with loess (STL) to determine the trend, seasonal and residual components, with the trend element being extracted as the main focus. Data cleaning was used to handle non-numeric values and formatting consistency [71]. Mann–Kendall analysis was then applied to the extracted trend component. The standardized Mann–Kendall Z-statistic was used to characterize the direction and standardized statistical evidence of monotonic AOT trends, with positive and negative Z-values indicating increasing and decreasing trends, respectively, while p-values were used to assess statistical significance at p < 0.05. Theil–Sen slopes were also calculated separately as estimates of trend magnitude. Fig 3 and S7 Table specifically present and summarize the location-level Mann–Kendall Z-statistics rather than the Theil–Sen slope estimates. Accordingly, the Z-statistic is dimensionless and should not be interpreted as the magnitude of AOT change. Together with STL decomposition, this approach provided an evaluation of long-term AOT trend patterns across CA.

In addition to trend analysis, heatwave events were first identified from the daily Tmax series using the Tx90-based definition described in Supplementary Section S2. The resulting heatwave information was then summarized at the monthly scale to match the temporal resolution of the monthly mean MERRA-2 AOT data. For each location and calendar month, HWN represents the number of heatwave events occurring during the month, HWF represents the total number of days belonging to heatwave events during the month, HWD represents the duration of the longest heatwave affecting that month, HWA represents the peak Tmax associated with the hottest heatwave during the month, and HWM represents the mean temperature associated with heatwave events during the month. Monthly mean AOT and the corresponding monthly heatwave indices were temporally aligned by location, year, and calendar month. Location-level correlations were then calculated using these temporally matched monthly AOT–heatwave-index pairs within each cluster, study period, and emission scenario to assess dust–heatwave co-variability. Because multiple correlation tests were performed across locations and heatwave indices, p-values were adjusted using the BH-FDR procedure at q = 0.05. Correlations with FDR-adjusted q-values below 0.05 were interpreted as statistically robust associations, while nominally significant correlations that did not remain significant after FDR correction were treated as suggestive but non-robust. This approach reduced the risk of overinterpreting false-positive correlations arising from multiple location-level tests [72].

A methodological consideration in this analysis is that Tmax was included as one of the meteorological predictors used by the LSTM to estimate AOT, together with Tmin, precipitation, and wind speed, while Tmax was also used to derive the heatwave indices. Tmax was retained in the AOT prediction framework because it provides meteorologically relevant predictive information and because the RF analysis showed that the relative predictive importance of the meteorological variables varied among clusters, indicating that AOT predictions were based on a multivariate set of meteorological controls rather than Tmax alone. Nevertheless, the shared use of Tmax introduces potential structural statistical dependence between modeled AOT and the heatwave indices. Therefore, the resulting AOT–heatwave correlations are interpreted as measures of statistical co-variability under shared meteorological conditions and not as evidence of an independent or causal relationship between dust and heatwaves.

## 3. Results

### 3.1. Statistical assessment of bias correction performance

S4 Table shows the statistical evaluation of the multi-model GCM ensemble for Tmax, Tmin, precipitation, and wind speed during the warm season in CA over the historical evaluation period. The bias-corrected ensemble exhibits reduced bias and RMSE relative to the unadjusted outputs, indicating improved agreement with ERA5 (S4 Table). RMSE was calculated from daily paired GCM-ensemble and ERA5 values at the evaluated locations and was subsequently averaged over the study domain. The reported RMSE therefore represents the domain-averaged daily paired error rather than an error accumulated as a function of the number of locations or observations. Temperature RMSE is reported in °C, precipitation RMSE in mm day^−1^, and wind-speed RMSE in m s^−1^. The relatively large residual temperature RMSE reflects remaining daily mismatch between the free-running GCM ensemble and ERA5 rather than the size of the evaluation sample. Although residual errors remain, the bias-corrected fields show improved agreement with the reanalysis benchmark relative to the uncorrected simulations (S4 Table).

### 3.2. The relationship between AOT and influencing parameters

S3 Fig depicts RF analysis of meteorological variables, such as 10-m wind speed, maximum 2-m temperature, minimum 2-m temperature, and precipitation focusing on their statistical association with the variations in AOT (Figs 1 and S3).

Here, FI (RF feature-importance) scores are used to indicate the relative predictive strength of each variable but not actual causation. Despite the existence of diverse geographic and climatic variations in the region, the analysis reveals which meteorological variables have the strongest predictive importance for AOT variability. Clusters 1 and 6 are specifically characterized by the high predictive importance of precipitation (31.37% and 30.76%, respectively) and 10-m wind speed (21.72% and 26.28%, respectively).

Cluster 2 shows a distinct profile, in which 10-m wind speed has the highest RF feature-importance score (32.88%), indicating that it is the dominant statistical predictor of AOT within this cluster. Clusters 3 and 4 also show strong predictive contributions from temperature and precipitation. The key influencing predictors in Cluster 3 are precipitation (26.80%) and 2-m maximum temperature (25.50%) whereas in Cluster 4, 2-m maximum temperature (29.98%) has the highest predictive importance.

Clusters 4 and 5 show a significant predictive contribution from maximum temperature at 2m with FI score of 29.98% and 31.65%, respectively. Moreover, minimum temperatures have FI scores of 26.75% and 29.52%, which indicates a strong association between temperature variability and AOT variability.

### 3.3. Changing patterns in heatwave indices

S5 Table shows heatwave-related indices, such as heatwave number (HWN), heatwave duration (HWD), heatwave frequency (HWF), heatwave amplitude (HWA), and heatwave magnitude (HWM), in CA for three 30-year climatological periods: historical (1992–2021), near-future (2022–2051), and far-future (2071–2100). These 30-year windows were selected to ensure robust statistics for extreme events such as heatwaves and dust storms under the SSP2–4.5 and SSP5–8.5 scenarios. Because Tx90 was calculated separately for each 30-year period and location, the heatwave indices reported here represent heat extremes relative to the contemporaneous climatology of each period. Accordingly, comparisons among periods describe changes in relative heatwave characteristics rather than exceedances above a fixed historical temperature threshold. Results show increases in several heatwave indices, with variability between clusters and scenarios. HWN shows increases across several clusters, especially under the high-emission SSP5–8.5 scenario. Even clusters with historically low HWN, like Cluster 6, show increases, indicating changes in the spatial distribution of relative heatwave occurrence (S5 Table).

HWD and HWF follow similar patterns. Cluster 2 shows prolonged heatwave conditions and near-continuous event frequency under SSP5–8.5 (S5 Table). HWA and HWM show marked increases across all clusters in terms of intensity, although the degree of these changes differs by region in the near-future. Cluster 6, which has historically exhibited elevated HWA values, is anticipated to surpass 36.98 °C under the SSP5–8.5 scenario in the far-future, whereas HWM in the same cluster may increase to 33.41 °C. In contrast, Cluster 2 is expected to experience HWA reaching 35.90 °C and HWM at 32.33 °C, indicating that while intensity increases, the overall impact is less severe than in Cluster 6. There is an increase in indices of frequency, duration of time and intensity across most clusters especially under SSP5–8.5. Overall, these changes vary among clusters, indicating spatially heterogeneous changes in heatwave characteristics (S5 Table).

S6 Table presents Sen’s slope and Mann–Kendall trend analyses of the heatwave indices. Sen’s slopes quantify the magnitude and direction of temporal change, while the percentage of locations with Mann–Kendall p-values < 0.05 indicates the spatial consistency of statistically significant trends within each cluster. Positive Sen’s slopes occur for several heatwave indices, particularly HWF, although the direction, magnitude, and spatial significance of the trends vary among indices, clusters, periods, and scenarios. Figs 2 and S4–S7 illustrate the spatial patterns of Sen’s slopes for HWD, HWF, HWN, HWM, and HWA from 1992 to 2100.

**Fig 2:**
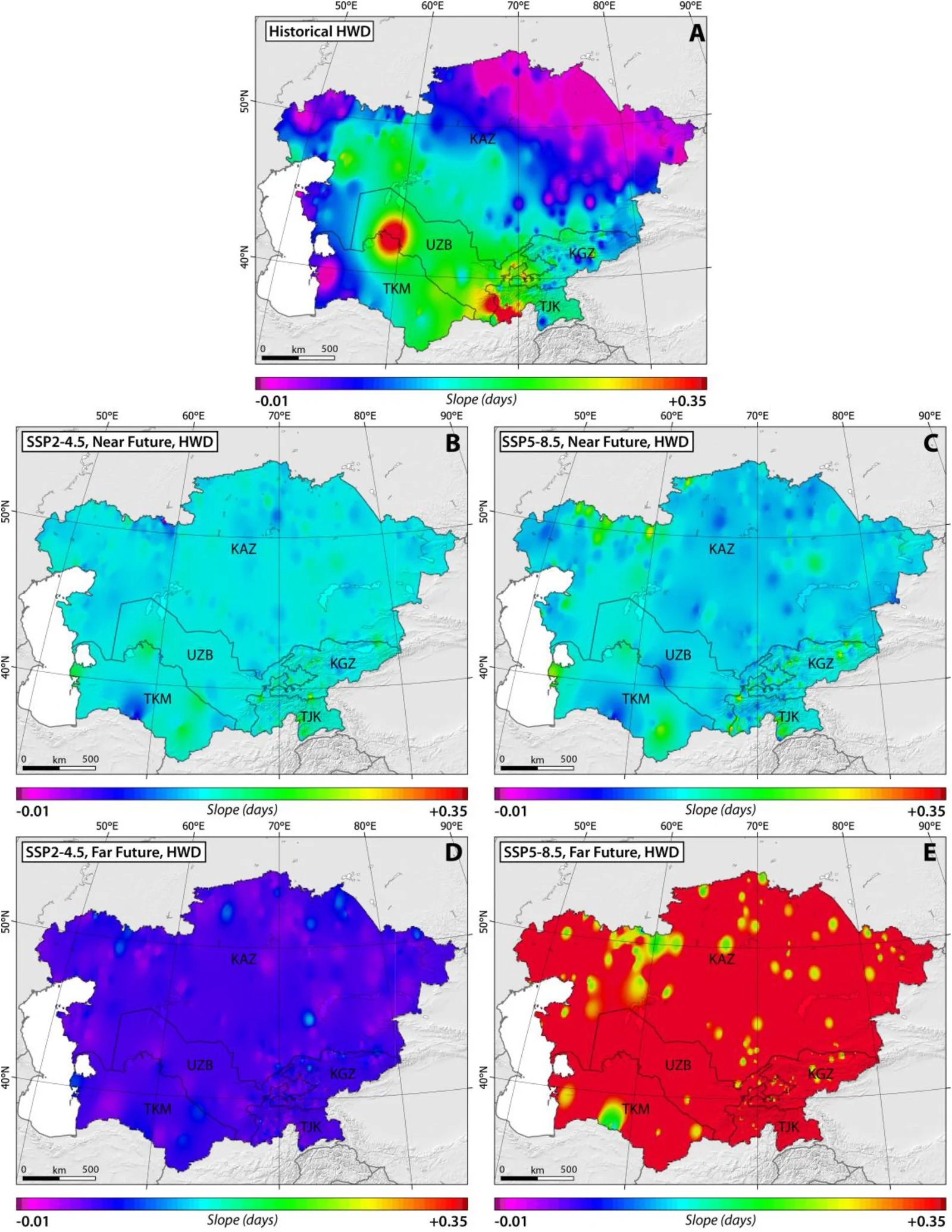
Spatial distribution of Sen’s slopes for heatwave duration (HWD) across CA from 1992 to 2100 under SSP2–4.5 and SSP5–8.5. The panels show the historical period (1992–2021), near-future SSP2–4.5 and SSP5–8.5 (2022–2051), and far-future SSP2–4.5 and SSP5–8.5 (2071–2100). Positive and negative Sen’s slopes indicate increasing and decreasing trends in HWD, respectively. Color-bar ranges are standardized across panels to facilitate comparison.

During the historical period (1992–2021), substantial spatial heterogeneity is evident among the heatwave indices. Cluster 1 shows small negative Sen’s slopes for HWD (−0.026) and HWN (−0.026), together with a small positive slope for HWF (0.029). In Cluster 2, HWF shows a comparatively strong positive slope (0.546), while HWA also increases (0.198); in contrast, HWN shows a small negative slope (−0.006). Clusters 3–6 generally show positive slopes for HWD, HWA, HWF, and HWM, with HWF displaying particularly strong increases: 0.613 in Cluster 3, 0.864 in Cluster 4, 0.442 in Cluster 5, and 0.960 in Cluster 6. HWN increases slightly in Clusters 3–5 (0.038, 0.023, and 0.001, respectively) but decreases slightly in Cluster 6 (−0.008). The Mann–Kendall results indicate that the spatial consistency of statistical significance differs substantially among indices; historical HWF trends, for example, are significant at a large proportion of locations in Clusters 2–6, whereas HWM generally exhibits much lower spatial significance (S6 Table; Figs 2; S4–S7).

In the near-future (2022–2051), Sen’s slopes are positive for all five heatwave indices across all clusters under both scenarios, although their spatial statistical significance differs among indices. Under SSP2–4.5, HWF slopes range from 0.628 to 0.658 across the six clusters, while HWA slopes range from 0.143 to 0.159. HWD also shows consistently positive slopes (0.136–0.142), and HWM ranges from 0.051 to 0.067. Although HWN slopes are positive (0.135–0.144), none of the locations within the clusters show statistically significant HWN trends at p < 0.05 under SSP2–4.5. By contrast, HWF trends are statistically significant at 100% of locations across all six clusters, while HWA, HWD, and HWM also show widespread spatial significance.

Under near-future SSP5–8.5, the magnitude of the positive HWF slopes increases substantially, ranging from 0.980 to 1.006, while HWA slopes range from 0.216 to 0.231 and HWM slopes from 0.116 to 0.134. Positive HWN slopes are also observed across all clusters (0.178–0.186), although statistically significant HWN trends occur at only a small proportion of locations. HWF and HWM, in contrast, show statistically significant trends at 100% of locations in every cluster. These results demonstrate that positive Sen’s slopes do not necessarily correspond to spatially widespread statistical significance and highlight differences among the individual heatwave metrics (S6 Table; Figs 2; S4–S7).

In the far-future (2071–2100), the two scenarios produce markedly different trend patterns. Under SSP2–4.5, HWF retains small positive Sen’s slopes across all clusters (0.203–0.211), while HWD and HWN also remain weakly positive. In contrast, HWA shows negative slopes across all clusters (−0.026 to −0.013), as does HWM (−0.024 to −0.017). These trends generally exhibit limited spatial statistical significance, with only small proportions of locations reaching p < 0.05.

Under far-future SSP5–8.5, much stronger positive Sen’s slopes emerge for HWF (1.421–1.475) and HWA (0.129–0.143) across all six clusters. HWF trends are statistically significant at 100% of locations in every cluster, while HWA trends are statistically significant at approximately 98.5–100% of locations. HWD and HWN also show positive Sen’s slopes, although their spatial statistical significance is comparatively limited. In contrast, HWM shows consistently negative Sen’s slopes across all clusters (−0.066 to −0.051), with statistically significant trends at 100% of locations. Because the heatwave thresholds are period-specific, these negative HWM trends should be interpreted as changes in relative heatwave magnitude within the corresponding future climatology rather than as evidence of declining absolute temperatures. Overall, the results indicate increasingly pronounced but index-dependent and scenario-dependent changes in heatwave characteristics across CA (S6 Table; Figs 2; S4–S7).

While heatwaves represent an important component of climate extremes in CA, atmospheric dust loading, represented here by dust extinction AOT, adds another dimension to these environmental risks. We therefore next examine the spatial and temporal evolution of AOT trends across the six clusters before evaluating dust–heatwave co-variability.

### 3.4. Changing patterns in AOT

We adopted trend and frequency analyses to identify temporal trends and changes in the frequency of different dust-intensity categories and to assess the stability or evolution of these patterns.

#### Mann–Kendall trend analysis.

Fig 3 presents the spatial distribution of Mann–Kendall Z-statistics for AOT trends across CA from 1992 to 2100 under the SSP2–4.5 and SSP5–8.5 scenarios, while S7 Table summarizes cluster-specific statistics of the location-level Mann–Kendall Z-values, including their maximum, minimum, mean, and standard deviation, together with the percentage of locations exhibiting statistically significant Mann–Kendall trends (p < 0.05) for the different study periods. The Mann–Kendall Z-statistic is dimensionless; positive Z-values indicate increasing monotonic trends and negative Z-values indicate decreasing monotonic trends. The sign of the Mann–Kendall Z-statistic indicates the direction of the monotonic trend, while its absolute magnitude reflects the standardized statistical evidence for that trend; it should not be interpreted as the magnitude of AOT change.

**Fig 3:**
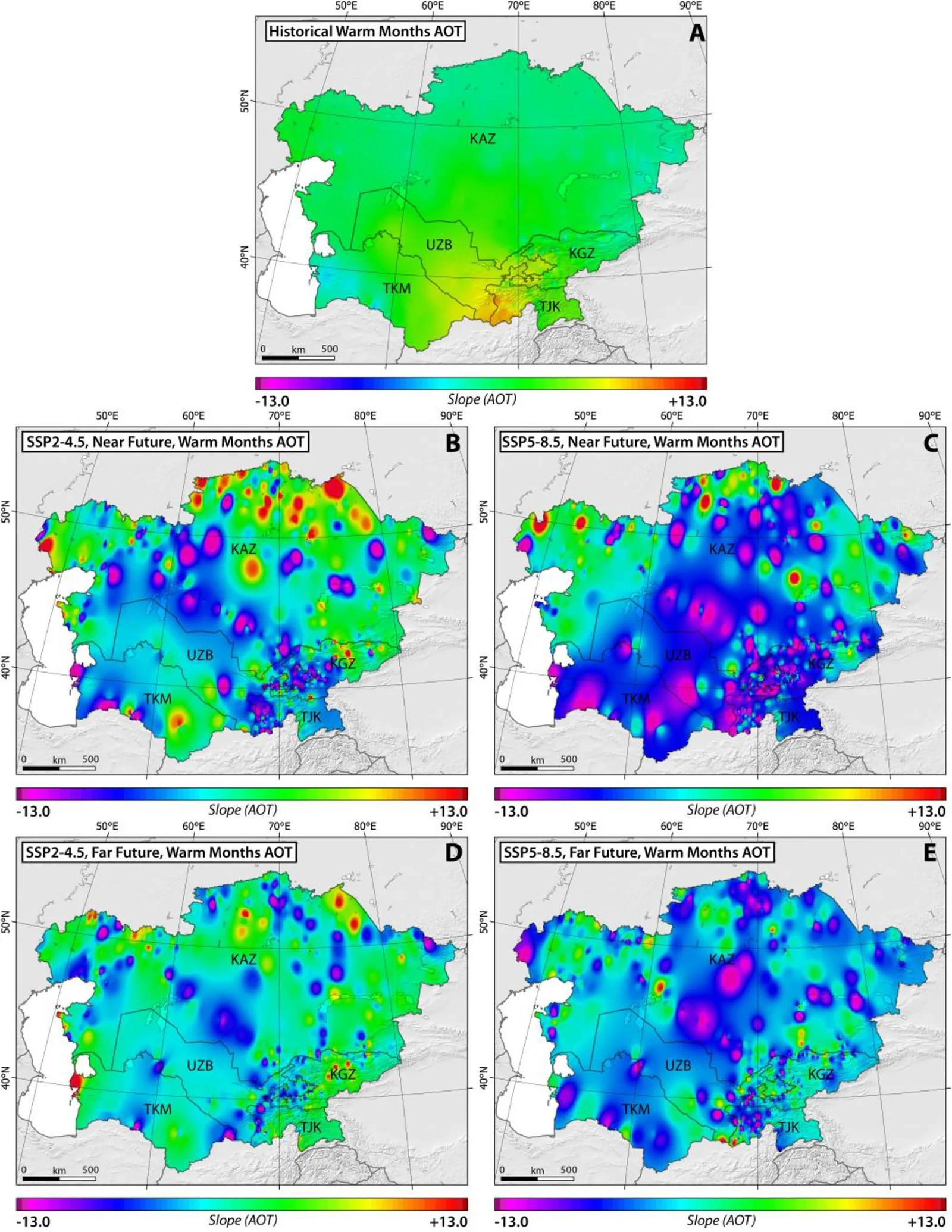
Spatial distribution of Mann–Kendall Z-statistics for AOT trends across CA during the warm season from 1992 to 2100 under SSP2–4.5 and SSP5–8.5. Positive Z-values indicate increasing AOT trends, whereas negative Z-values indicate decreasing AOT trends. The Mann–Kendall Z-statistic is dimensionless.

During the historical period, the mean Mann–Kendall Z-statistics differed substantially among the six clusters, indicating spatial heterogeneity in the predominant direction of AOT trends. Positive mean Z-values were observed in Cluster 1 (1.53), Cluster 2 (0.58), Cluster 4 (4.17), and Cluster 5 (5.65), whereas Cluster 3 showed a slightly negative mean Z-value (−0.16) and Cluster 6 remained close to neutral (0.01). The standard deviation of the location-level Z-statistics was 1.28 in Cluster 1, 0.71 in Cluster 2, 0.27 in Cluster 3, 3.97 in Cluster 4, 1.66 in Cluster 5, and 1.06 in Cluster 6, indicating differences in the spatial variability of trend statistics among clusters (S7 Table and Fig 3).

Under the near-future SSP2–4.5 scenario (2022–2051), the location-level Mann–Kendall Z-statistics show greater within-cluster variability than during the historical period. Cluster 1 remains close to neutral, with a mean Z-statistic of 0.07 and a standard deviation of 7.01. Negative mean Z-statistics are observed in Clusters 2 (−1.77), 4 (−2.37), 5 (−5.71), and 6 (−1.93), whereas Cluster 3 shows a positive mean Z-statistic of 3.18. Among the six clusters, Cluster 5 exhibits the most negative mean Z-statistic (−5.71), indicating the strongest predominance of decreasing AOT trends, whereas Cluster 3 exhibits the most positive mean Z-statistic (3.18), indicating a predominance of increasing trends. The standard deviation of the location-level Z-statistics ranges from 5.15 in Cluster 6 to 8.05 in Cluster 4, demonstrating substantial spatial heterogeneity in the trend statistics within individual clusters (S7 Table and Fig 3).

By the far-future period (2071–2100) under SSP2–4.5, negative mean Mann–Kendall Z-statistics are observed in most clusters. Cluster 1 is the exception, with a slightly positive mean Z-statistic of 0.32 and a standard deviation of 5.54. Mean Z-statistics are negative in Cluster 2 (−0.59; standard deviation = 6.04), Cluster 3 (−0.84; standard deviation = 5.59), Cluster 4 (−0.90; standard deviation = 6.21), Cluster 5 (−1.38; standard deviation = 6.28), and Cluster 6 (−1.37; standard deviation = 5.82). These results indicate a predominance of decreasing AOT trends across these clusters, although the variability of the location-level Z-statistics demonstrates substantial spatial heterogeneity (S7 Table and Fig 3).

Under near-future SSP5–8.5 (2022–2051), negative mean Mann–Kendall Z-statistics are observed across all six clusters. The mean Z-statistics are −4.65, −1.24, −3.67, −5.53, −8.05, and −5.14 in Clusters 1–6, respectively. Cluster 5 therefore exhibits the most negative mean Z-statistic (−8.05), indicating the strongest predominance of decreasing AOT trends among the six clusters. The corresponding standard deviations range from 6.17 to 7.93, demonstrating considerable spatial heterogeneity in the location-level trend statistics (S7 Table and Fig 3).

Similarly, under far-future SSP5–8.5 (2071–2100), negative mean Mann–Kendall Z-statistics persist across all six clusters, with mean values of −3.26, −2.30, −4.17, −1.81, −2.62, and −3.65 in Clusters 1–6, respectively. Cluster 3 exhibits the most negative mean Z-statistic (−4.17), followed by Cluster 6 (−3.65) and Cluster 1 (−3.26). These results indicate that declining AOT trends predominate across the clusters under the far-future SSP5–8.5 scenario, although the magnitude and spatial variability of the standardized trend statistics differ among clusters (S7 Table and Fig 3).

Overall, the Mann–Kendall analysis demonstrates substantial spatial and scenario-dependent heterogeneity in AOT trends. The historical period is characterized by positive mean Z-statistics in several clusters, whereas future conditions, particularly under SSP5–8.5, are characterized predominantly by negative mean Z-statistics. These Z-statistics describe the direction and standardized statistical evidence of monotonic AOT trends rather than the magnitude of AOT change. To complement these trend patterns, we also analyzed dust-intensity frequencies to examine changes in the distribution of dust-event magnitudes.

#### Dust intensity frequency analysis.

S8 Table shows the frequencies of low-, moderate-, high-, and very-high-intensity dust events across the six clusters during the historical, near-future, and far-future periods under SSP2–4.5 and SSP5–8.5. For historical-period, the percentage contribution of each intensity category is calculated relative to the total number of dust events within that cluster. In S8 Table, the percentage columns for future periods represent the frequency of each intensity category relative to the historical frequency of the same category within the same cluster, with the historical value defined as 100%. These calculations are over fixed 30-year periods under each climate scenario. This provides a consistent basis for comparing category-specific changes between the historical and future periods.

Historically, Cluster 1 was characterized by a substantial number of low-intensity events (5,196 occurrences, 21.4% of all historical events in the cluster) and moderate-intensity events (16,576 occurrences, 68.1%). Under near-future SSP2–4.5, the frequency of low-intensity events decreases to 924, corresponding to 17.8% of the historical low-intensity frequency, while under SSP5–8.5 it decreases to 1,130, corresponding to 21.7% of the historical value. High and very high intensities are projected to nearly vanish. By the far-future, moderate-intensity events reach 104.5% and 102.8% of their historical frequency under SSP2–4.5 and SSP5–8.5, respectively, whereas low-intensity events remain below half of their historical frequency (S8 Table).

Clusters 2 and 3 show scenario- and intensity-dependent changes in dust-event frequencies. In Cluster 2, low-intensity events fall to 3,012 (48.3% of the historical low-intensity frequency) under near-future SSP2–4.5, while high and very high intensities are substantially reduced, with very-high-intensity events falling to zero. Cluster 3, which historically experienced many low (11,402) and moderate (3,282) intensity events, shows strong near-future reductions, particularly under SSP2–4.5, when low- and moderate-intensity frequencies decline to 40.1% and 5.8% of their respective historical frequencies. By the far-future, however, the total number of events across all intensity categories is 11,780 under both scenarios, corresponding to 80.0% of the historical total of 14,725 events. Cluster 4 historically recorded 1,703 moderate-, 2,027 high-, and 4,640 very-high-intensity events, corresponding to 20.3%, 24.2%, and 55.4% of all historical events in the cluster, respectively. Future changes in Cluster 4 differ by period and scenario; notably, very-high-intensity frequencies exceed their historical level in the far-future, reaching 128.9% under SSP2–4.5 and 125.6% under SSP5–8.5. Cluster 5 historically recorded substantial moderate (20,339, 56.8%) and high (9,626, 26.9%) intensity levels. Future changes in Cluster 5 are also intensity-dependent: under far-future SSP5–8.5, moderate-intensity events remain below their historical frequency (78.7%), whereas high-intensity events slightly exceed the historical frequency (106.7%) (S8 Table).

Since both heatwaves and dust events often occur under similar synoptic conditions, the following section examines their statistical covariance and assesses how their relationship evolves under future scenarios.

### 3.5. Correlation between AOT levels and heatwave related indices

Figs 4–8 illustrate the spatial and temporal distribution of the correlation between heatwave-related indices and AOT from 1992 to 2100 under SSP2–4.5 and SSP5–8.5. S9 Table provides a comprehensive analysis of the positive and negative correlations between dust extinction AOT and heatwave indices (HWM, HWA, HWN, HWD, HWF) across six distinct clusters in CA. Results show pronounced variability across space, time, and emission scenarios. These correlations reflect statistical co-variability rather than direct causation, as both heatwaves and AOT levels can be driven by common synoptic conditions, such as persistent high-pressure systems, aridity, and wind regimes. Our analysis aims to assess the strength of these concurrent patterns rather than infer causal mechanisms.

**Fig 4:**
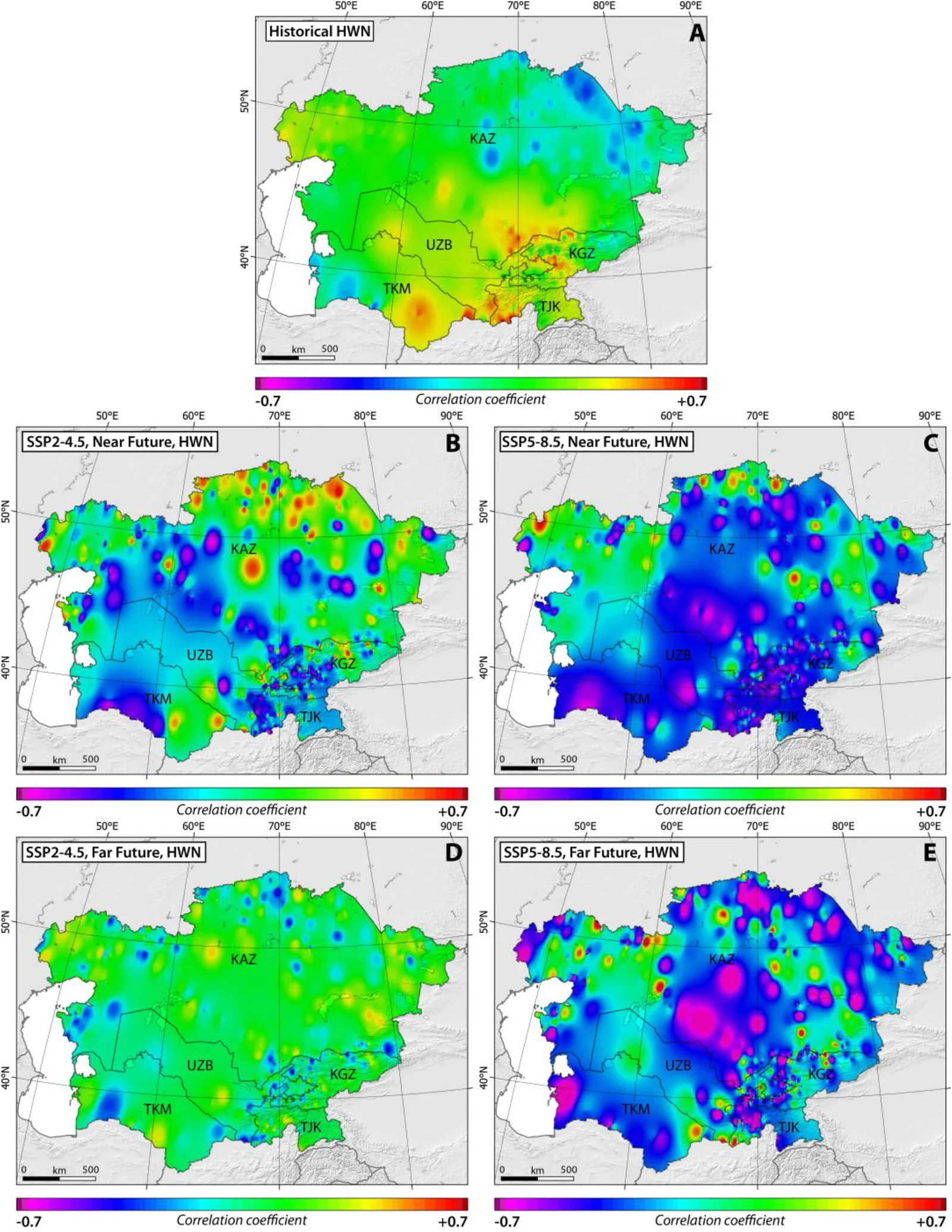
Spatiotemporal distribution of location-level correlations between HWN and AOT across CA from 1992 to 2100 under SSP2–4.5 and SSP5–8.5.

**Fig 5:**
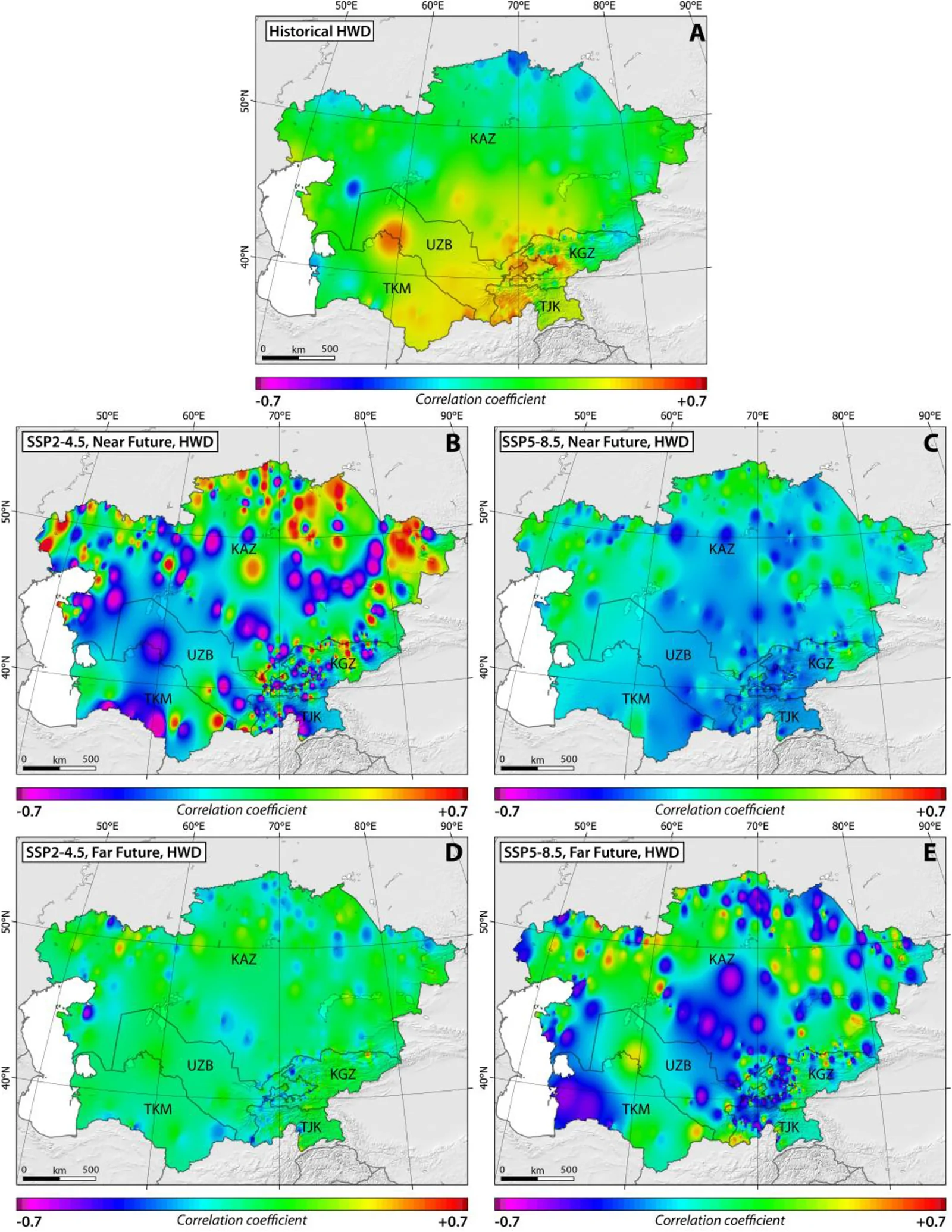
Spatiotemporal distribution of location-level correlations between HWD and AOT across CA from 1992 to 2100 under SSP2–4.5 and SSP5–8.5.

**Fig 6:**
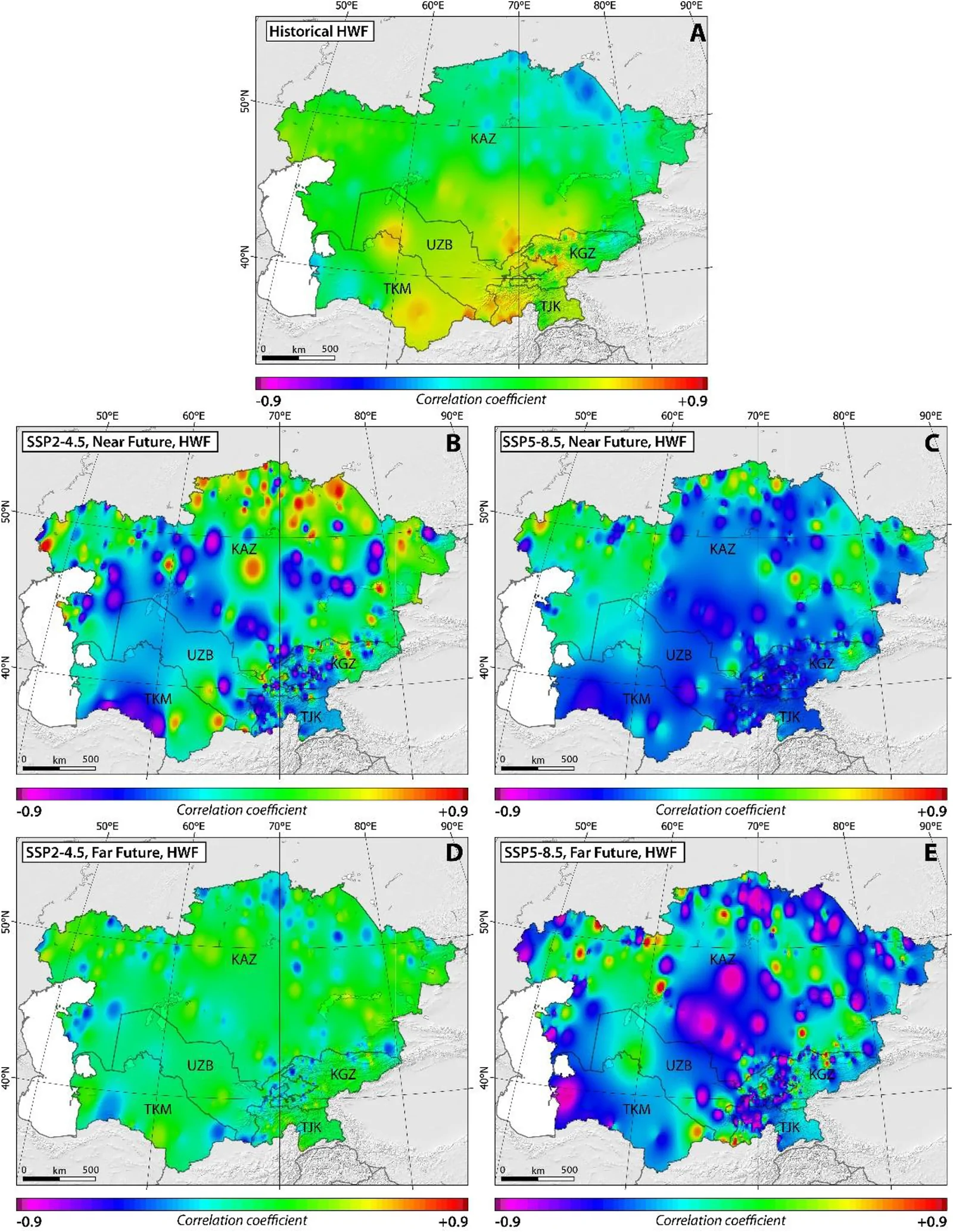
Spatiotemporal distribution of location-level correlations between HWF and AOT across CA from 1992 to 2100 under SSP2–4.5 and SSP5–8.5.

**Fig 7:**
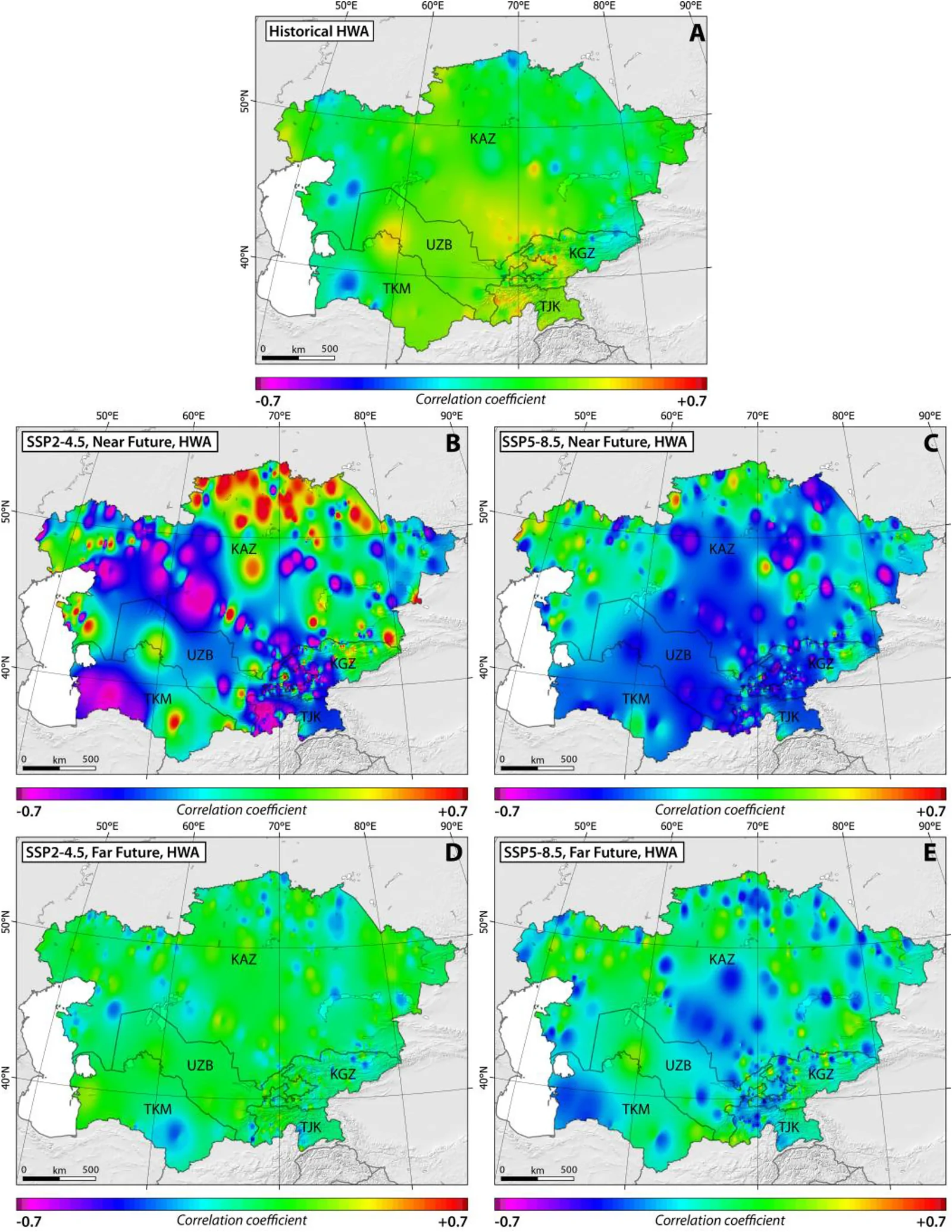
Spatiotemporal distribution of location-level correlations between HWA and AOT across CA from 1992 to 2100 under SSP2–4.5 and SSP5–8.5.

**Fig 8:**
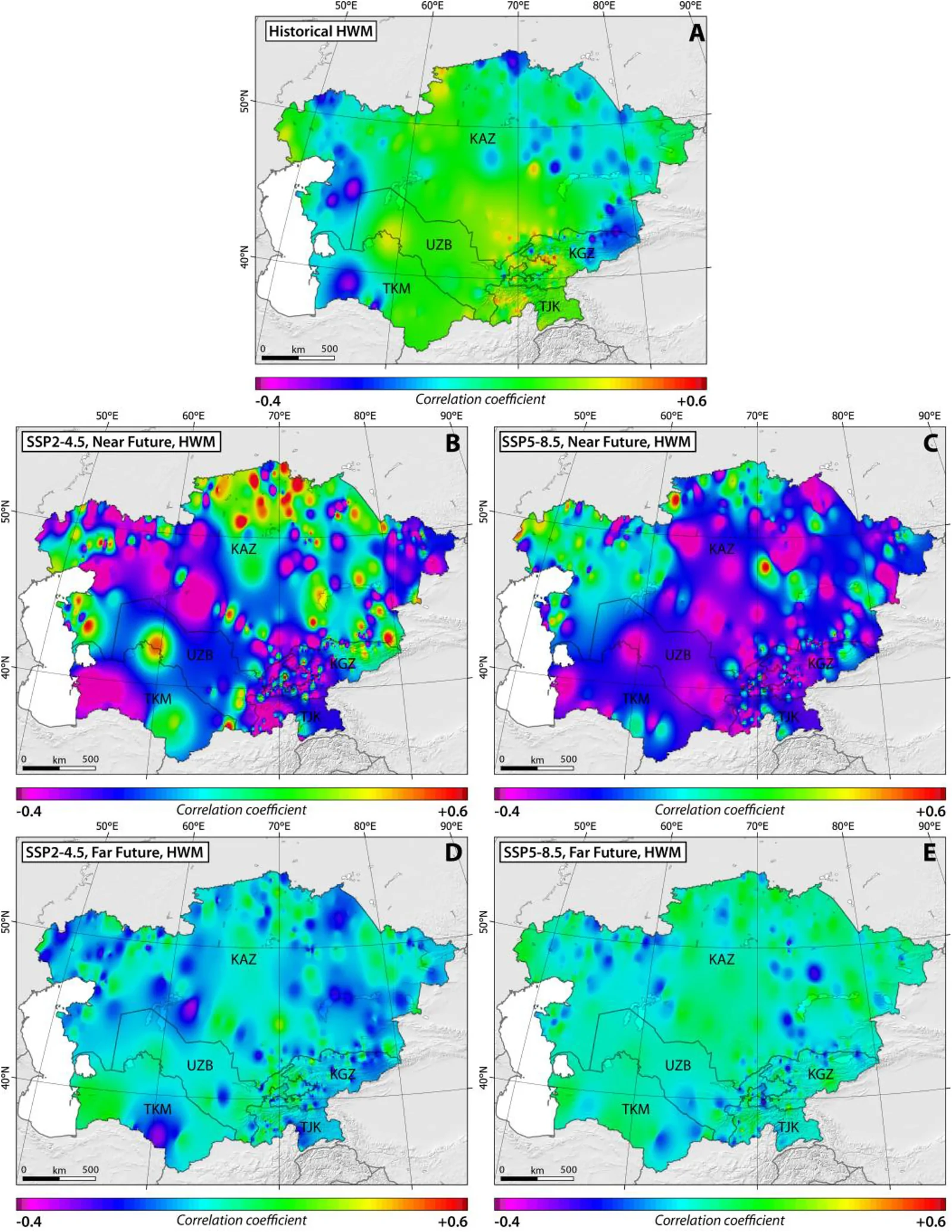
Spatiotemporal distribution of location-level correlations between HWM and AOT across CA from 1992 to 2100 under SSP2–4.5 and SSP5–8.5.

To account for multiple location-level correlation tests within each cluster, period, scenario, and heatwave index, p-values were further adjusted using BH-FDR procedure at q = 0.05. Therefore, significant correlations are interpreted only where they remained FDR-significant after multiple-comparison correction. Correlations that were nominally significant at p < 0.05 but did not remain significant after BH-FDR correction were treated as suggestive but statistically non-robust. Overall, FDR-corrected results confirm that dust–heatwave co-variability is spatially heterogeneous and concentrated in specific cluster–index combinations rather than being regionally uniform. Correlations vary by cluster – from strong positive to strong negative – indicating that there is no consistent cluster-scale compound dust–heatwave signature.

After BH-FDR correction, only a subset of the nominally significant location-level correlations remained statistically robust. Among the 150 cluster–period–scenario–index combinations assessed, 44 retained at least one FDR-significant location. The strongest retained associations are summarized in S10 Table. Historically, the most robust signal was observed for HWF in Cluster 6, where 68.0% of locations remained significant after FDR correction. Additional strong FDR-supported relationships were identified for HWA and HWM under SSP2–4.5, particularly in Clusters 1, 2, and 5. These results indicate that the most robust dust–heatwave co-variability is associated mainly with heatwave frequency and intensity metrics rather than all heatwave indices equally.

Clusters 3, 1, and 5 exhibit the largest variability in correlation patterns. Cluster 3 shows wide variability in its association with HWD, with correlations from −0.83 to 0.83 in the near-future (SSP2–4.5), narrowing to −0.61 to 0.57 in the far-future (SSP5–8.5). HWA correlations range from −0.77 to 0.85 historically, shrinking to −0.43 to 0.42, suggesting weakening associations over time. Cluster 1 shows increasing HWF correlations, from −0.18 to 0.64 historically to −0.88 to 0.88 under SSP5–8.5, but HWA remains highly variable (historical: −0.27 to 0.47; future: −0.59 to 0.44), reflecting instability (S9 Table and Figs 4−8). Cluster 5 shows the widest variability, e.g., HWD (−0.84 to 0.85) and HWF (−0.88 to 0.90). Cluster 2 is more stable: HWN correlations rise from 0 to 0.45 historically, then span −0.77 to 0.83 in the far-future. HWA highly variable, spanning both negative and positive values (−0.92 to 0.84). Cluster 4 fluctuates but stabilizes later. Cluster 6 shows the least variability, with narrow ranges across all metrics (e.g., HWM: −0.21 to 0.19 in the far-future) (S9 Table and Figs 4–8). Overall, these mixed signs indicate localized and scenario-dependent co-variability rather than a spatially coherent dust–heatwave mechanism across all clusters.

In the historical period (1992–2021), several clusters exhibited nominally high positive correlations; however, after BH-FDR correction, the most statistically robust patterns were concentrated in HWF. Cluster 6 showed the strongest FDR-supported association with HWF, with 68.0% of locations remaining significant, followed by Cluster 5 for HWF (58.0%) and Cluster 1 for HWF (53.8%). In the near-future period under SSP2–4.5, the strongest FDR-supported relationships shifted toward heatwave amplitude and magnitude. Cluster 1 showed a robust association with HWA, with 61.1% of locations remaining significant after FDR correction. Cluster 5 also showed a strong FDR-supported association with HWA (58.4%), while Cluster 2 retained strong FDR-supported relationships with HWA (51.6%) and HWM (48.4%). In the far-future and under SSP5–8.5, fewer relationships remained FDR-significant, suggesting that several nominal correlations were not sufficiently robust after multiple-comparison correction. Although several SSP5–8.5 cluster–index combinations retained FDR-significant locations, none ranked among the strongest FDR-supported associations summarized in S10 Table, indicating weaker retained statistical support under high-emission conditions.

Overall, historical FDR-supported patterns were strongest for HWF, particularly in Clusters 6, 5, and 1, while near-future SSP2–4.5 relationships were strongest for HWA and HWM, especially in Clusters 1, 2, and 5. Under SSP5–8.5, fewer nominal correlations remained FDR-significant. Taken together, the BH-FDR results support a cautious interpretation: AOT and heatwave indices show localized and scenario-dependent co-variability, but they do not support a single coherent cluster-scale compound dust–heatwave signal across CA.

## 4. Discussion

### 4.1. Assessment of bias-corrected model performance

The residual temperature RMSE after bias correction reflects the remaining daily mismatch between the bias-corrected GCM ensemble and ERA5 across the study domain. Because RMSE was calculated from paired daily modeled and ERA5 values at the evaluated locations and subsequently averaged over the study domain, its magnitude does not arise from accumulation with the number of locations or observations. The remaining errors are more plausibly associated with the difficulty of reproducing local-scale daily temperature variability across the climatically and topographically heterogeneous Central Asian domain using relatively coarse-resolution GCM simulations. In general, the raw output of general circulation models (GCMs) tends to overestimate climate variability in the interior regions of continents due to their relatively coarse spatial resolution. In contrast, the bias correction process reduces these discrepancies by adjusting the statistical distribution of the model data to be more consistent with the observational data.

As for precipitation, the relatively low RMSE values can be explained by the predominantly arid climatic conditions of CA, where precipitation is often very low or close to zero for a significant part of the warm season. The equal-weighted multi-model ensemble may also reduce model-specific variability, although the performance statistics themselves were calculated using daily paired values rather than monthly aggregated data. Overall, the better agreement between the corrected precipitation fields and the reanalysis reference data indicates that the bias correction framework used is suitable for assessing regional climate.

### 4.2. Climatic Drivers of AOT Variability in CA

The variables identified in the feature importance analysis using the random forest (RF) model mainly represent statistical relationships between parameters and do not necessarily indicate direct causal relationships. However, many of the meteorological variables highlighted in this analysis are physically known to be associated with the behavior and variability of aerosols in the atmosphere. Precipitation can remove suspended particles from the atmosphere through the process of wet deposition, thereby reducing the optical thickness of aerosols. In contrast, independent dust-physics studies have shown that wind speed can contribute to dust-particle mobilization and regional or long-range transport, especially in the arid and semi-arid regions of CA, which are prone to dust production [73–76].

The observed differences between different clusters also indicate that the interactions between the land surface and the atmosphere do not follow a uniform pattern across CA. For example, cluster 6 mainly includes high-altitude areas in the Altai and Tianshan mountain ranges, while cluster 1 mainly includes lowland and irrigated areas with different surface characteristics. Although both clusters are statistically associated with changes in precipitation and wind speed, the processes controlling these relationships are likely to be different due to differences in topography, land cover, and climatic conditions. In some regions, especially in cluster 2, wind speed has higher predictive importance for AOT than the other meteorological predictors considered here. In addition, temperature-related variables can also be statistically associated with dust extinction AOT through their relationships with surface drying, atmospheric stability, boundary-layer development, and vertical mixing processes [77]. Meanwhile, heavy precipitation events also play an important role in removing aerosol particles from the atmosphere by enhancing the wet deposition process [78–80].

Overall, the findings of this study indicate that aerosol variability in CA is statistically associated with local meteorological conditions. Accounting for these regionally varying meteorological controls may improve statistical representation and prediction of dust extinction AOT across CA. Such an approach, in addition to increasing the predictive ability of models, can also play an effective role in environmental management planning and developing climate change adaptation strategies [73,77,81].

### 4.3. Regional heterogeneity and drivers of heatwave changes

The increases in heatwave intensity and amplitude indices across different clusters indicate changes in relative temperature extremes across CA. Because the Tx90 threshold was recalculated for each 30-year period and location, these changes are defined relative to the contemporaneous climatology and should not be interpreted solely as absolute exceedances above the historical thermal baseline. However, the magnitude and rate of these changes are not the same in all clusters, indicating substantial spatial heterogeneity in projected heatwave characteristics (S5 Table). In general, scenarios with higher greenhouse gas emissions show stronger responses in heatwave indices. The magnitude of projected change nevertheless differs among clusters and indices, particularly under SSP2–4.5, demonstrating that the response of relative heatwave characteristics to future warming is spatially heterogeneous rather than uniform [82]. Such patterns suggest that the response of the climate system to moderate emission conditions is not necessarily linear and behaves differently in different regions.

The projected strengthening of several period-specific heatwave indices across multiple clusters suggests that relative heat extremes may become more prominent across a broader part of CA under future climate scenarios. Accordingly, adaptation planning should account for the cluster-specific combinations of increasing heatwave frequency, duration, and intensity rather than assuming a uniform regional response. Strengthening regional resilience depends largely on local-scale adaptation capacities, as well as the development of higher spatial resolution models that can simulate topographic effects, land–atmosphere feedback, and regional atmospheric circulation processes with greater accuracy [30,83]. One possible mechanism that could lead to increased heatwave intensity in mountainous areas is the reduction of glacier and snow cover under high greenhouse gas emission scenarios such as the SSP5–8.5 scenario. Reduced ice cover reduces the surface albedo and, as a result, increases the absorption of solar radiation, intensifying surface warming in high-elevation areas, a process that could lead to increased temperature extremes in high-altitude areas that were previously moderated by snow and ice cover [84].

Studies conducted in the Middle East and North Africa (MENA) region also provide a useful comparison, as these regions share significant arid and semi-arid climate characteristics with CA. According to previous studies, the duration of heatwaves in historical conditions has typically been reported to be around 4–6 days, but in future scenarios this duration could increase to more than 10 days and even reach several weeks by the end of the century in high-emission scenarios [85]. These estimates are broadly consistent with the direction of the increasing trend in heatwave indices observed in this study for CA, although direct quantitative comparison should be made cautiously because heatwave definitions and reference thresholds may differ among studies.

Similar evidence has also been reported for Africa, where Adigun et al. [86] projected increases in heatwave magnitude, number, duration, and frequency under future climate scenarios using the Excess Heat Factor framework. Their results indicated that heatwave duration may lengthen substantially by the end of the 21^st^ century, with stronger increases under higher-emission pathways, and that heat-related mortality could also increase markedly under future warming. This comparison strengthens the cross-regional interpretation that arid and semi-arid regions are likely to experience increasing heatwave persistence and severity under continued climate change [86].

### 4.4. Interpretation of AOT trend patterns and implications

Combined analysis of Mann–Kendall trend statistics and dust-intensity frequencies provides complementary information on the future evolution of atmospheric dust loading. This approach also allows us to examine how the distribution of dust event intensity is evolving by identifying areas where persistent or emerging AOT changes are observed. Together, these analyses characterize both the predominant direction of monotonic AOT trends and changes in the frequency distribution of dust-intensity categories across CA.

The existing variations between clusters show that the dynamics of dust in this area do not have a homogeneous pattern. This heterogeneity indicates that the varying land-atmosphere interaction regimes across CA make differences in dust release and transport processes. The differences among clusters demonstrate that AOT trends do not follow a homogeneous spatial pattern across CA. The Mann–Kendall Z-statistics presented in Fig 3 and S7 Table characterize the direction and standardized statistical evidence of monotonic AOT trends, whereas the separately calculated Theil–Sen slopes quantify trend magnitude. Importantly, the Z-statistic is dimensionless and should not be interpreted as the magnitude of AOT change. Moreover, monotonic-trend statistics summarize the overall tendency within each analysis period and may not capture nonlinear evolution, abrupt changes, or reversals within the time series. Such behavior is plausible because atmospheric dust loading is sensitive to interacting with changes in precipitation, surface conditions, and atmospheric circulation.

The positive mean Mann–Kendall Z-statistic in Cluster 3 under near-future SSP2–4.5 indicates a predominance of increasing monotonic AOT trends within this cluster. Climatic conditions such as changes in aridity, surface moisture, and wind regimes could contribute to such behavior, although the present statistical analysis does not isolate the causal mechanism. In contrast, the negative mean Z-statistics in Clusters 5 and 6 indicate a predominance of decreasing monotonic AOT trends during the near-future periods. Such changes could be associated with meteorological conditions that reduce atmospheric dust loading, including changes in precipitation, surface moisture, or transport, but the present model does not explicitly simulate dust-source emissions. The negative Mann–Kendall Z-statistics in the far-future SSP5–8.5 projections do not indicate negative AOT concentrations; rather, they indicate a predominance of decreasing monotonic AOT trends. Overall, the different behavior of the clusters suggests that the response of dust to climate change in CA has a heterogeneous spatial pattern. However, the present analysis was conducted at the cluster scale and therefore does not explicitly isolate individual dust-source regions. For example, the Aralkum, one of the major anthropogenic dust-source areas in CA, is embedded within a broader regional cluster. Consequently, future changes in its spatial extent, surface properties, and emission potential cannot be separately evaluated and may contribute additional uncertainty to projected AOT trends.

The two scenarios of emission SSP2–4.5 and SSP5–8.5 also reflect the importance of greenhouse gas emission pathways in developing future dust dynamics. From a risk-management perspective, areas characterized by increasing AOT trends may warrant greater attention for dust-related preparedness and exposure reduction. Conversely, decreasing AOT trends indicate lower meteorology-associated atmospheric dust loading under the modeled conditions, but they should not be interpreted directly as reductions in dust-source emissions. Large-scale atmospheric circulation can also modulate both heat extremes and atmospheric dust loading. Persistent blocking, anomalous high-pressure systems, atmospheric stability, and changes in ventilation or transport pathways may influence the co-occurrence of heat and dust; however, the direction of this relationship is not uniform across CA, as demonstrated by the mixed positive and negative correlations observed in this study [87]. The nuanced concept of these processes is significant in the creation of local adaptation processes and the control of the ecological and health impact of altered dust concentrations.

### 4.5. Interpretation of dust intensity trends and emission pathway effects

The intensity frequency change analysis indicates that in the majority of Central Asian clusters there exists a tendency toward a reduction in high- and very-high-intensity dust events, but the magnitude of this decline varies regionally and across different emission scenarios. Although reductions in high- and very-high-intensity events occur in several cluster–period combinations, the response is not uniform across CA or consistently ordered between SSP2–4.5 and SSP5–8.5. For example, some clusters retain or exceed historical frequencies of specific high-intensity categories in the far-future.

The trend of changes is also not the same in clusters. Clusters 1 and 5 depict the most diverse variation in the frequency of dust intensity which might reflect a greater sensitivity of these regions to different greenhouse gas emission pathways. Clusters 2 and 3 exhibit different category-specific responses from Clusters 1, 4, and 5, further emphasizing that changes in dust-event frequency depend on cluster, intensity class, period, and scenario. Conversely, cluster 4 has high levels of dust intensity across both scenarios. This trend indicates that geographical conditions or local environmental characteristics might determine the dominant role in the continuance of dust activity in this area.

In certain clusters, notably under high emission conditions, very high intensity dust events persist, pointing to the sustained susceptibility of these areas to severe dust episodes. Therefore, a clearer insight into the dynamics of dust within each cluster may be highly significant for planning target adaptation and risk-mitigation strategies in areas most affected by severe dust events. Overall, the results demonstrate that greenhouse-gas emission pathways influence projected dust-intensity distributions, but the response is spatially heterogeneous and does not show a simple monotonic relationship in which the lower-emission scenario consistently produces fewer extreme dust events across all clusters.

A noteworthy result is the projected reduction, and in some cluster–period combinations near-disappearance, of high- and very-high-intensity dust events under the future scenarios. Given the expected warming and increasing aridity in parts of CA, this pattern may initially appear counterintuitive. Several factors may contribute to this result. First, the LSTM-based framework was trained using historical meteorological–AOT relationships and may have limited ability to extrapolate rare extreme AOT values beyond the range represented in the training data; this is consistent with broader concerns that data-driven Earth-system models require process understanding when applied outside historical conditions [88]. Second, projected changes in precipitation and surface moisture may locally suppress dust mobilization despite increasing temperatures, especially where moisture availability offsets enhanced evaporative demand. Previous dust-cycle studies show that soil moisture, surface erodibility, wind, and land-surface conditions strongly regulate dust emission and transport [38,89]. Third, future land-surface changes, including vegetation responses and changes in dust-source characteristics, may alter dust-emission potential in ways not explicitly represented in the present framework. Recent studies also show that increased vegetation cover and soil moisture can contribute to reduced dust activity [90]. Therefore, the projected reduction in extreme dust-event frequency should be interpreted cautiously and may reflect a combination of climatic, ecological, and methodological factors rather than a single controlling mechanism.

### 4.6. Interpretation and implications of dust–heatwave co-variability

In CA, heatwaves and atmospheric dust do not have a uniform relationship, and their association is spatially heterogeneous and scenario-dependent. Due to the specific conditions among different clusters, the interaction between these two phenomena takes place with different ranges of correlation coefficients and signs.

After applying BH-FDR correction to the location-level correlation tests, only selected cluster–index combinations retained statistically robust associations, while many nominally significant correlations did not remain significant after controlling for multiple comparisons. Positive AOT–heatwave correlations occur at some locations, whereas other locations show weak or negative relationships. Therefore, the correlation results should not be interpreted as evidence of a single coherent compound dust–heatwave mechanism across the whole region. Instead, they indicate localized co-variability that may differ among clusters, periods, and emission scenarios. This localized co-variability may partly reflect shared synoptic and land–atmosphere conditions, which influence their respective processes, such as persistent high-pressure systems, suppressed precipitation, enhanced aridity, atmospheric stability, and surface-wind variability; all of which can raise air temperature and also favor dust transport and atmospheric dust loading. Recent work on arid regions of Asia and Africa further emphasizes that atmospheric evaporative demand can intensify drought-related moisture stress, especially where warming increases reference evapotranspiration and accelerates soil-moisture depletion. Ogunrinde et al. [91] showed that evaporative-demand-based drought indicators can reveal stronger drying tendencies than precipitation-only indices in arid environments, highlighting the importance of temperature, humidity, wind speed, and radiation in controlling land–atmosphere moisture stress. These mechanisms are relevant to CA because enhanced evaporative demand and aridity can contribute to surface drying, heatwave persistence, and conditions favorable for localized dust–heatwave co-variability [91].

The large variance in cluster 5 indicates that both local land-atmosphere interactions and regional dust-transport conditions are relevant in determining the relationship between dust and heatwaves in this area. After FDR correction, robust signals in Cluster 5 were mainly retained for specific heatwave intensity-related indices, particularly HWA in the near-future SSP2–4.5 period, rather than across all heatwave metrics. This suggests that the dust–heatwave linkage in this cluster is more index-specific than uniform. The presence of both strongly positive and strongly negative correlations within some clusters further suggests that local synoptic variability, transport pathways, and sampling variability may partly influence the observed relationships.

The FDR-corrected results also indicate that the most robust dust–heatwave associations were concentrated mainly in HWF, HWA, and HWM. Historically, the strongest retained signals were associated with HWF, especially in Clusters 6, 5, and 1. In the near-future SSP2–4.5 scenario, robust associations shifted toward HWA and HWM, particularly in Clusters 1, 2, and 5. By contrast, under SSP5–8.5, many nominally significant correlations became non-significant after FDR adjustment, implying that high-emission projections may produce more spatially fragmented and less statistically robust dust–heatwave correlation patterns.

From a climate risk management perspective, the behavioral differences between clusters highlight the importance of developing targeted adaptation strategies in high emission scenarios. Measures including dust-source management where feasible, heatwave preparedness, exposure reduction, and climate-resilient planning may therefore be appropriate depending on local conditions. In addition, some ecosystem-based measures can also play an important role in reducing risks; For example, ecosystem-based measures, including vegetation restoration and land-management interventions where locally appropriate, may also contribute to reducing dust-related vulnerability [92]. Such approaches are compatible with multi-hazard climate risk management frameworks, as these frameworks emphasize the simultaneous management of climate extremes that interact with each other, rather than considering each hazard in isolation. Nevertheless, adaptation priorities should be based on the specific local patterns of heatwave intensification and dust-related atmospheric variability, rather than assuming a uniform compound hazard across all clusters.

The coincidence of dust events and heatwaves is of considerable importance from a public health perspective, in addition to its environmental consequences. Prolonged exposure to very high temperatures can increase the risk of heat-related illnesses and cardiovascular stress [52,93,94]. At the same time, increased dust concentrations in the air put additional strain on the respiratory system and can exacerbate diseases such as asthma and bronchitis caused by exposure to particulate matter [95–99]. Therefore, areas where elevated atmospheric dust loading coincides with high heatwave frequency, amplitude, or magnitude may be more vulnerable, especially in densely populated areas or areas where the population is at higher risk. However, based on the FDR-corrected correlation results, the strongest statistically robust overlap appears to involve HWF, HWA, and HWM more consistently than HWD alone.

These patterns highlight the importance of designing integrated preparedness strategies; strategies that simultaneously manage heat and dust risks. Measures such as dust control and mitigation, heatwave preparedness programs, and the development of resilient infrastructure can be effective in this regard. Also, the development of early warning systems and improved air quality monitoring networks can strengthen regional health and climate preparedness capacity. At the same time, intervention priorities should be determined according to the characteristics of each cluster; for example, regions showing simultaneous increases in heatwave frequency, amplitude, or magnitude and positive AOT associations may require integrated heat-health and air-quality preparedness, whereas regions with weak or negative associations may require separate hazard-specific planning.

### 4.7. Uncertainties and limitations

Although climate models and shared socio-economic pathways (SSP) scenarios provide valuable tools for estimating future trends in AOT, dust activity, and heatwaves, they are still subject to uncertainties. These uncertainties are mainly due to assumptions about socio-economic development pathways, structural limitations of climate models, and incomplete understanding of the interactions between dust and heatwaves in the climate system.

ERA5 reanalysis data are used as a reference source in many climate studies due to their relatively high spatial resolution and wide coverage. However, this dataset may also have regional biases in some regions, especially in regions with poor observational data or significant geographical complexity, such as parts of CA. In this region, the lack of dense, long-term observational networks limits the ability to fully evaluate model output. For this reason, ERA5 data were used in this study alongside the MERRA-2 dataset to provide a more reliable reference frame for the analysis by leveraging multiple global observational sources as the reference dataset for evaluating and bias-correcting the climate variables, while MERRA-2 provided the historical dust extinction AOT data used for the aerosol analysis and LSTM training. However, the extracted indices and the model calibration process may still be affected by possible biases uncertainties in either reanalysis product may propagate into the derived heatwave indices, model evaluation, and AOT-based analyses. To partially reduce these uncertainties, the CMIP6 model outputs were aligned with the ERA5 data after bias correction, using a consistent bias-correction framework across the analyzed periods [100,101].

Bias correction methods are usually based on the assumption that the difference between the model-simulated values and the observed data remains constant over time. However, this assumption may not always hold in situations where the climate system is changing rapidly. As a result, such a limitation can affect the estimates of AOT and heat wave indices, introducing uncertainties in their intensity and spatial distribution. In this study, a hybrid semiparametric bias correction method was used because it is able to adjust the entire statistical distribution of climate variables, thereby providing a more accurate representation of average conditions and extreme events. The “one model, one vote” averaging method used to combine model outputs does not explicitly consider the structural dependencies among some of the models in the CMIP6 ensemble. In practice, a number of these models may be based on similar development frameworks or closely related parameterization schemes. Such similarities can reduce the degree of true independence of the members of the model ensemble and thus influence the final estimates [102]. Although the use of equal weighting is common in many studies and is consistent with previous research [60], approaches that consider kinship or dependency relationships between models can provide a more accurate picture of the uncertainties. In addition, the relatively coarse spatial resolution of many CMIP6 models prevents them from fully representing climate processes at local scales, an issue that becomes more important in areas with complex topography or diverse land uses.

An additional methodological consideration concerns the use of period-specific heatwave thresholds. In this study, the Tx90 threshold was calculated separately for each 30-year study period and location. Consequently, future heatwave indices represent extremes relative to the contemporaneous temperature distribution of each period rather than exceedances above a fixed historical thermal threshold. The projected changes in HWN, HWD, HWF, HWA, and HWM should therefore be interpreted as changes in relative heatwave characteristics under each climatological period and not solely as the effect of absolute background warming. A fixed historical Tx90 threshold would provide a complementary assessment of future exceedance of historical heating conditions and could result in different magnitudes of projected change. Comparison of period-specific and fixed historical thresholds was beyond the scope of the present study and should be considered in future work to further evaluate the sensitivity of projected heatwave characteristics to threshold selection.

On the other hand, the performance of the deep learning LSTM model used in this study for prediction is not uniform across regions. The RMSE value across the studied cities ranges from 0.024 to 0.109; the average value is estimated to be around 0.057, and the standard deviation is estimated to be 0.019. In most cities, the error value falls between the 25^th^ percentile (0.041) and the 75^th^ percentile (0.070). In contrast, higher error values in some regions are likely related to differences in data quality, spatial heterogeneity of dust distribution, or difficulty in representing complex spatial interactions.

A further limitation is that the LSTM-based future AOT estimates rely on the assumption that the historical relationship between meteorological predictors and MERRA-2 dust extinction AOT remains sufficiently stable under future climate conditions. In this study, AOT represents atmospheric dust loading, not explicit dust-source emissions. Therefore, the LSTM framework captures statistical associations between temperature, precipitation, wind speed, and atmospheric dust extinction AOT, but it does not explicitly simulate future dust-source activation, land-cover change, vegetation change, soil properties, soil-moisture memory, agricultural shifts, or SSP-specific anthropogenic aerosol-emission inventories. This limitation is particularly relevant for localized and evolving dust-source regions such as the Aralkum. Because the analysis was conducted at the cluster scale, future changes in the extent, surface characteristics, and emission potential of individual dust-source regions could not be resolved explicitly. Consequently, changes associated with the continued evolution of the dried Aral Sea bed are not directly represented and remain an important source of uncertainty in the projected regional AOT response. Accordingly, future AOT projections should be interpreted as scenario-conditioned estimates of meteorology-associated atmospheric dust loading rather than deterministic projections of future dust emissions.

An additional limitation of the dust–heatwave correlation analysis arises from the shared use of Tmax within the analytical framework. Tmax was included as one of the meteorological predictors used by the LSTM to estimate AOT, together with Tmin, precipitation, and wind speed, while Tmax was also used to derive the heatwave indices. This shared predictor introduces potential structural statistical dependence between modeled AOT and the heatwave metrics, such that part of the observed correlation may reflect their common dependence on temperature-related meteorological variability. Although the LSTM estimates AOT from multiple meteorological predictors and the relative predictive importance of these variables varies among clusters, the present analysis cannot fully separate this shared-predictor effect from the observed dust–heatwave co-variability. Therefore, the reported correlations should be interpreted as statistical co-variability under shared meteorological conditions rather than as evidence of an independent or causal relationship between dust and heatwaves. A sensitivity analysis using an independently trained and evaluated AOT model excluding Tmax would provide a useful assessment of the magnitude of this structural dependence and should be considered in future work.

In addition, the RF feature-importance analysis was based on the mean decrease in impurity criterion, which may introduce bias in predictor ranking, particularly when predictor variables differ in variability or are correlated with each other. Therefore, the reported feature-importance values should be interpreted as indicative rankings of predictive relevance rather than exact measures of variable influence. Future work should integrate dynamic land-surface information, source-specific dust-emission schemes, and aerosol outputs from Earth-system models to better represent the combined influence of meteorology, land-surface change, and emission pathways on dust-related hazards in CA.

## 5. Conclusion

This study examines atmospheric dust variability and its statistical co-variability with heatwave indices across CA. The results provide a comprehensive assessment of the future behavior of the two phenomena under SSP2–4.5 and SSP5–8.5. Our findings indicated that dust events and heatwaves in CA show regionally heterogeneous statistical co-variability. However, dust variations are not homogeneous throughout the region. Both regional climatic conditions and greenhouse-gas emission pathways influence the magnitude and direction of these changes. The cluster-based analysis confirms that different parts of CA respond differently to climate change. Depending on the climate scenario, some parts show increasing AOT trends, whereas others remain close to neutral or exhibit decreasing trends by the end of the 21^st^ century.

Correlation analysis also showed that the relationship between atmospheric dust and heatwave indices is spatially variable and scenario-dependent. After applying the BH-FDR correction to location-level correlation tests, only selected cluster–index combinations retained statistically robust associations, confirming that many nominal correlations were not sufficiently robust after accounting for multiple comparisons. The strongest FDR-supported relationships were mainly associated with HWF, HWA, and HWM, indicating that dust–heatwave co-variability is more strongly linked to heatwave frequency and intensity characteristics than to all heatwave metrics equally.

Under SSP5–8.5, some clusters exhibit positive dust–heatwave associations, while others show weak or negative associations, highlighting the spatial heterogeneity of these relationships. Moreover, the period-specific heatwave indices indicate increases in heatwave frequency and persistence over time in several clusters. These changes represent relative heat extremes within the corresponding climatological periods rather than exceedances above a fixed historical temperature threshold. Together with the spatially heterogeneous AOT patterns, these results suggest that the potential for localized co-occurrence of prolonged heatwaves and elevated atmospheric dust may increase in selected regions under high-emission scenarios. However, the correlation analysis does not support a uniform compound dust–heatwave signal across CA.

Comparison of SSP2–4.5 and SSP5–8.5 shows that the future evolution of heatwave and dust-related conditions is strongly scenario- and region-dependent. Higher-emission conditions are associated with stronger changes in several heatwave indices, whereas the AOT response remains spatially heterogeneous, with both increasing and decreasing trends across clusters. Consequently, the likelihood and character of overlapping heatwave and dust-related atmospheric risks cannot be described by a single regional response and should instead be evaluated at the cluster and local scales. Where elevated heatwave conditions coincide with enhanced atmospheric dust loading, the combined exposure may increase environmental and public-health risks, particularly in densely populated or otherwise vulnerable areas.

Our study, from a methodological perspective, is one of the first attempts to assess the interaction between heatwaves and atmospheric dust comprehensively in CA using a multi-model ensemble of CMIP6 models, regional clustering analysis, and machine learning techniques under different climate scenarios. Our results indicate that greenhouse gas emission pathways play an important role in shaping future climate hazards. In addition, the analytical framework presented here can support the identification of regions exhibiting localized heatwave–dust co-variability and can inform air-quality management, climate-resilient land-use planning, and the development of multi-hazard early warning systems. Nevertheless, further studies are required to better understand the long-term implications of co-occurring dust and heatwave conditions, particularly their combined effects on human health and ecosystem stability. Future work should also incorporate dynamic land-surface processes, source-specific dust emissions, and potential feedbacks between heat, aridity, atmospheric circulation, and dust to improve predictive capability and support region-specific adaptation and mitigation planning.

The projections presented in this study remain subject to uncertainty arising from SSP assumptions, structural differences among CMIP6 models, limitations of the bias-correction framework, and uncertainty in machine-learning-based AOT projections. Consequently, the findings should be interpreted as plausible scenario-conditioned future pathways rather than deterministic predictions. Further improvements in regional observations, climate-model resolution, dust-process representation, and physically informed data-driven modelling will be important for reducing these uncertainties.

## Supporting information

S1 Table List of provinces in CA with corresponding numbers and country codes. (DOCX)

S2 Table The list of GCM models used in the current study (https://www.ipcc.ch/report/ar6). (DOCX)

S3 Table Climate indices, their definitions, and units used in this study. Heatwave indices (HWN, HWD, HWF, HWA, HWM) are calculated based on daily maximum temperature (T_max_) exceeding the 90^th^ percentile threshold (Tx90), determined using a 15-day moving window.
(DOCX)

S4 Table Statistical evaluation of bias-correction performance for Tmax, Tmin, precipitation, and wind speed during the historical evaluation period. RMSE was calculated using daily paired GCM-ensemble and ERA5 values at the evaluated locations and subsequently averaged over the study domain. RMSE is reported in °C for Tmax and Tmin, mm day^−1^ for precipitation, and m s^−1^ for wind speed.
(DOCX)

S5 Table The statistics of calculated climate indices across CA, divided into six clusters, under two climate scenarios include the sum for HWN, HWF, and HWD, and the average for HWA and HWM between 1992 and 2100. (DOCX)

S6 Table Sen’s slope and Mann–Kendall trend analysis of heatwave-related indices during the historical (1992–2021), near-future (2022–2051), and far-future (2071–2100) periods under SSP2–4.5 and SSP5–8.5 across the six clusters in CA. The reported trend values are Sen’s slopes, which quantify the magnitude and direction of temporal change. Mann–Kendall p-values were evaluated at each location, and the percentage rows report the proportion of locations within each cluster with statistically significant trends (p < 0.05), indicating the spatial consistency of trend significance.
(DOCX)

S7 Table Mann–Kendall Z-statistic analysis of AOT trends across CA, divided into six clusters, during the historical, near-future, and far-future periods under SSP2–4.5 and SSP5–8.5. The table reports the maximum, minimum, mean, and standard deviation of location-level Mann–Kendall Z-statistics within each cluster. Positive Z-values indicate increasing monotonic trends and negative Z-values indicate decreasing monotonic trends. The Z-statistic is dimensionless. The percentage of locations with statistically significant Mann–Kendall trends (p < 0.05) is also reported for each cluster.
(DOCX)

S8 Table Dust intensity frequencies and percentage changes across CA, divided into six clusters, under historical and future climate scenarios (SSP2–4.5 and SSP5–8.5) during warm periods between 1992 and 2100. (DOCX)

S9 Table The range of positive and negative correlations, including observed maximum and minimum correlations, between dust levels and heatwave indices (HWM, HWA, HWN, HWD, HWF) for six clusters across historical and future scenarios (SSP2–4.5 and SSP5–8.5) in CA between 1992 and 2100. (DOCX)

S10 Table Strongest FDR-supported dust–heatwave associations across Central Asia. (DOCX)

S1 Fig Diagnostics used for selecting the optimal number of clusters in the K-means analysis. (A) Elbow and silhouette diagnostics showing the variation of within-cluster sum of squares (WCSS) and silhouette score as a function of the number of clusters (*k*). The vertical dashed line indicates the selected solution (*k* = 6), where the reduction in WCSS begins to diminish while maintaining a relatively high silhouette score. (B) Percentage reduction in WCSS relative to the previous cluster number. The reduction decreases substantially after *k* = 6, indicating limited additional improvement from further partitioning.
(PNG)

S2 Fig The overview of the LSTM network structure which is used in the current research. (PNG)

S3 Fig The percentages of the features included in RF during the studied period (1992–2021) across clusters in CA. (PNG)

S4 Fig The slope of spatial-temporal changes of HWF in CA, between 1992 and 2100 under climate scenarios of SSP2–4.5 and SSP5–8.5. Each panel presents five plots corresponding to Historical, SSP2–4.5 near-future, SSP5–8.5 near-future, SSP2–4.5 far-future, and SSP5–8.5 far-future, allowing direct visual comparison of temporal and scenario-based patterns.
(PNG)

S5 Fig The slope of spatial-temporal changes of HWN in CA, between 1992 and 2100 under climate scenarios of SSP2–4.5 and SSP5–8.5. Each panel presents five plots corresponding to Historical, SSP2–4.5 near-future, SSP5–8.5 near-future, SSP2–4.5 far-future, and SSP5–8.5 far-future, allowing direct visual comparison of temporal and scenario-based patterns.
(PNG)

S6 Fig The slope of spatial-temporal changes of HWM in CA, between 1992 and 2100 under climate scenarios of SSP2–4.5 and SSP5–8.5. Each panel presents five plots corresponding to Historical, SSP2–4.5 near-future, SSP5–8.5 near-future, SSP2–4.5 far-future, and SSP5–8.5 far-future, allowing direct visual comparison of temporal and scenario-based patterns.
(PNG)

S7 Fig The slope of spatial-temporal changes of HWA in CA, between 1992 and 2100 under climate scenarios of SSP2–4.5 and SSP5–8.5. Each panel presents five plots corresponding to Historical, SSP2–4.5 near-future, SSP5–8.5 near-future, SSP2–4.5 far-future, and SSP5–8.5 far-future, allowing direct visual comparison of temporal and scenario-based patterns.
(PNG)
