# Supplementary material for: Climate change-driven compound dust storm and heatwave extremes in Central Asia under future CMIP6 scenarios
Source: PLoS One. 2026 Nov 4;21(11):e0359347. doi: 10.1371/journal.pone.0359347 (PMC13734455; doi:10.1371/journal.pone.0359347)
Supplement: Supplementary file 1 [file pone.0359347.s001.docx]

| **Table S1.** List of provinces in CA with corresponding numbers and country codes. | | |
| --- | --- | --- |
| **Kazakhstan** | | |
| ***Number*** | ***Region*** | ***Country code*** |
| 1 | Almaty | KAZ |
| 2 | Aqmola | KAZ |
| 3 | Aqtöbe | KAZ |
| 4 | Atyrau | KAZ |
| 5 | East Kazakhstan | KAZ |
| 6 | Mangghystau | KAZ |
| 7 | North Kazakhstan | KAZ |
| 8 | Pavlodar | KAZ |
| 9 | Qaraghandy | KAZ |
| 10 | Qostanay | KAZ |
| 11 | Qyzylorda | KAZ |
| 12 | South Kazakhstan | KAZ |
| 13 | West Kazakhstan | KAZ |
| 14 | Zhambyl | KAZ |
| **Kyrgyzstan** | | |
| 15 | Batken | KGZ |
| 16 | Chuy | KGZ |
| 17 | Jalal-Abad | KGZ |
| 18 | Naryn | KGZ |
| 19 | Osh | KGZ |
| 20 | Talas | KGZ |
| 21 | Ysyk-Köl | KGZ |
| **Tajikistan** | | |
| 22 | Gorno-Badakhshan | TJK |
| 23 | Khatlon | TJK |
| 24 | Leninabad | TJK |
| 25 | Tadzhikistan Territories | TJK |
| 26 | Ahal | TKM |
| 27 | Balkan | TKM |
| 28 | Chardzhou | TKM |
| 29 | Mary | TKM |
| 30 | Tashauz | TKM |
| **Uzbekistan** | | |
| 31 | Andijon | UZB |
| 32 | Bukhoro | UZB |
| 33 | Ferghana | UZB |
| 34 | Jizzakh | UZB |
| 35 | Karakalpakstan | UZB |
| 36 | Kashkadarya | UZB |
| 37 | Khorezm | UZB |
| 38 | Namangan | UZB |
| 39 | Navoi | UZB |
| 40 | Samarkand | UZB |
| 41 | Sirdaryo | UZB |
| 42 | Surkhandarya | UZB |
| 43 | Tashkent | UZB |
