# Supplementary material for: Climate change-driven compound dust storm and heatwave extremes in Central Asia under future CMIP6 scenarios
Source: PLoS One. 2026 Nov 4;21(11):e0359347. doi: 10.1371/journal.pone.0359347 (PMC13734455; doi:10.1371/journal.pone.0359347)
Supplement: Supplementary file 2 [file pone.0359347.s002.docx]

| **Table S2.** The list of GCM models used in the current study ([https://www.ipcc.ch/report/ar6](https://www.ipcc.ch/report/ar6/wg1/downloads/report/IPCC_AR6_WGI_AnnexII.pdf)). | | |
| --- | --- | --- |
| MODELS | Resolution(km) and Number of Levels (L) | Institution/Country |
| ACCESS-CM2 | 140 km, 85 L | csiro-access csiro and Australia. res. council center of excellence for climate system science, Australia |
| BCC-CSM2-MR | 100 km, 46 L | BCC, Beijing Climate Centre, China |
| CESM2 | 100 km | NCAR, National Center for Atmospheric Research, USA |
| FGOALS-f3-L | 90 km, 32 L | CAS, Chinese Academy of Sciences, China |
| GFDL-ESM4 | 100 km, 49 L | NOAA-GFDL, National Oceanic and Atmospheric Administration, Geophysical Fluid Dynamics Laboratory, USA |
| IPSL-CM6A-LR | 160 km, 79 L | IPSL, Institute Pierre- Simon Laplace, France |
| MIROC6 | 250 km, 40 L | MIROC Consortium JAMSTEC, AORI, NIES, R-CCS, Japan |
| MIROC-ES2L | 250 km, 40 L | MIROC Consortium JAMSTEC, AORI, NIES, R-CCS, Japan |
| MPI-ESM1-2-LR | 170 km, 47 L | MPI-M, Max Planck Institute for Meteorology, Germany |
| MRI-ESM2-0 | 100 km, 80 L | MRI, Meteorological Research Institute, Japan |
