# Supplementary material for: Climate change-driven compound dust storm and heatwave extremes in Central Asia under future CMIP6 scenarios
Source: PLoS One. 2026 Nov 4;21(11):e0359347. doi: 10.1371/journal.pone.0359347 (PMC13734455; doi:10.1371/journal.pone.0359347)
Supplement: Supplementary file 3 [file pone.0359347.s003.docx]

| **Table S3.** Climate indices, their definitions, and units used in this study. Heatwave indices (HWN, HWD, HWF, HWA, HWM) are calculated based on daily maximum temperature (T_max_) exceeding the 90^th^ percentile threshold (Tx90), determined using a 15-day moving window. | | | | |
| --- | --- | --- | --- | --- |
| **ID** | **Index Name** | **Definition** | **Unit** | **Sectors of Economics** |
| HWD-Tx90 | Heatwave duration as defined by 90^th^ percentile of TX. | The length of the longest heatwave identified by HWN. | days | Health, Agriculture and Food Security, Water Resources and Hydrology |
| HWA-Tx90 | Heatwave amplitude as defined by the 90^th^ percentile of TX. | The peak daily value in the hottest heatwave (defined as the heatwave with highest HWM). | °C | Health, Agriculture and Food Security, Water Resources and Hydrology |
| HWF-Tx90 | Heatwave frequency as defined by the 90^th^ percentile of TX. | The number of days that contribute to heatwaves as identified by HWN. | days | Health, Agriculture and Food Security, Water Resources and Hydrology |
| HWN-Tx90 | Heatwave number as defined by 90^th^ percentile of TX. | The number of individual heatwaves that occur each summer (Nov – Mar in southern hemisphere and May – Sep in northern hemisphere). | events | Health, Agriculture and Food Security, Water Resources and Hydrology |
| HWM-Tx90 | Heatwave magnitude as defined by the 90^th^ percentile of TX. | The mean temperature of all heatwaves identified by HWN. | °C | Health, Agriculture and Food Security, Water Resources and Hydrology |
