# Supplementary material for: Climate change-driven compound dust storm and heatwave extremes in Central Asia under future CMIP6 scenarios
Source: PLoS One. 2026 Nov 4;21(11):e0359347. doi: 10.1371/journal.pone.0359347 (PMC13734455; doi:10.1371/journal.pone.0359347)
Supplement: Supplementary file 4 [file pone.0359347.s004.docx]

| **Table S4.** Statistical evaluation of bias and daily paired RMSE for maximum and minimum temperature, precipitation, and wind speed from raw and bias-corrected equal-weighted GCM ensemble simulations against ERA5 over Central Asia during 1992–2021. Error statistics were calculated at the evaluated locations and then averaged over the study domain. Temperature errors are reported in °C, precipitation errors in mm day⁻¹, and wind-speed errors in m s⁻¹. | | |
| --- | --- | --- |
| ***Maximum 2-m temperature*** | | |
|  | **Bias** | **RMSE** |
| Raw multi-ensembled GCM models | 2.90 | 17.68 |
| Bias-corrected multi-ensembled GCM models | 0.00 | 16.39 |
| ***Minimum 2-m temperature*** | | |
|  | **Bias** | **RMSE** |
| Raw multi-ensembled GCM models | -6.92 | 17.62 |
| Bias-corrected multi-ensembled GCM models | 0.00 | 16.15 |
| ***Precipitation*** | | |
|  | **Bias** | **RMSE** |
| Raw multi-ensembled GCM models | 1.00 | 2.40 |
| Bias-corrected multi-ensembled GCM models | 0.00 | 1.50 |
| ***10-m Wind speed*** | | |
|  | **Bias** | **RMSE** |
| Raw multi-ensembled GCM models | 0.10 | 1.34 |
| Bias-corrected multi-ensembled GCM models | 0.00 | 0.41 |
