# Supplementary material for: Climate change-driven compound dust storm and heatwave extremes in Central Asia under future CMIP6 scenarios
Source: PLoS One. 2026 Nov 4;21(11):e0359347. doi: 10.1371/journal.pone.0359347 (PMC13734455; doi:10.1371/journal.pone.0359347)
Supplement: Supplementary file 5 [file pone.0359347.s005.docx]

| **Table S5.** The statistics of calculated climate indices across CA, divided into six clusters, under two climate scenarios include the sum for HWN, HWF, and HWD, and the average for HWA and WHM between 1992 and 2100. | | | | | | | | | | | | | |
| --- | --- | --- | --- | --- | --- | --- | --- | --- | --- | --- | --- | --- | --- |
| ***Historical Period (1992-2021)*** | | | | | | | | | | | | | |
|  | **Cluster** | **HWN** | **HWD** | **HWF** | **HWA** | **WHM** |  | **Cluster** | **HWN** | **HWD** | **HWF** | **HWA** | **WHM** |
|  | 1 | 5,562 | 12,390 | 23,716 | 27.41 | 24.64 |  | 1 | 5,562 | 12,390 | 23,716 | 27.41 | 24.64 |
|  | 2 | 12,497 | 31,934 | 61,670 | 28.37 | 25.98 |  | 2 | 12,497 | 31,934 | 61,670 | 28.37 | 25.98 |
|  | 3 | 5,060 | 11,168 | 25,095 | 30.44 | 27.3 |  | 3 | 5,060 | 11,168 | 25,095 | 30.44 | 27.3 |
|  | 4 | 2,436 | 5,707 | 12,192 | 32.67 | 29.89 |  | 4 | 2,436 | 5,707 | 12,192 | 32.67 | 29.89 |
|  | 5 | 10,134 | 22,899 | 44,830 | 24.41 | 22.07 |  | 5 | 10,134 | 22,899 | 44,830 | 24.41 | 22.07 |
|  | 6 | 2,486 | 5,597 | 13,034 | 35.42 | 32.52 |  | 6 | 2,486 | 5,597 | 13,034 | 35.42 | 32.52 |
| ***Near-Future (2022-2051)*** | | | | | | | | | | | | | |
| ***SSP2–4.5*** | **Cluster** | **HWN** | **HWD** | **HWF** | **HWA** | **WHM** | ***SSP5–8.5*** | **Cluster** | **HWN** | **HWD** | **HWF** | **HWA** | **WHM** |
|  | 1 | 5,755 | 9,742 | 23,360 | 28.54 | 26.13 |  | 1 | 6,774 | 13,402 | 33,564 | 29.99 | 26.49 |
|  | 2 | 10,947 | 18,918 | 44,741 | 29.22 | 26.89 |  | 2 | 13,449 | 26,569 | 67,079 | 30.68 | 27.23 |
|  | 3 | 3,938 | 6,991 | 16,216 | 28.69 | 26.3 |  | 3 | 4,900 | 9,938 | 24,460 | 30.07 | 26.74 |
|  | 4 | 1,946 | 3,326 | 7,922 | 28.47 | 26.09 |  | 4 | 2,305 | 4,562 | 11,440 | 29.99 | 26.48 |
|  | 5 | 8,996 | 15,258 | 36,527 | 29.22 | 26.9 |  | 5 | 10,979 | 21,594 | 54,854 | 30.67 | 27.16 |
|  | 6 | 1,678 | 2,895 | 6,841 | 30.26 | 27.93 |  | 6 | 2,086 | 4,156 | 10,398 | 31.72 | 28.29 |
| ***Far-Future (2071-2100)*** | | | | | | | | | | | | | |
| ***SSP2–4.5*** | **Cluster** | **HWN** | **HWD** | **HWF** | **HWA** | **WHM** | ***SSP5–8.5*** | **Cluster** | **HWN** | **HWD** | **HWF** | **HWA** | **WHM** |
|  | 1 | 5,247 | 12,460 | 24,579 | 30.61 | 28.34 |  | 1 | 8,343 | 17,289 | 44,656 | 35.38 | 31.8 |
|  | 2 | 10,270 | 23,786 | 47,628 | 31.37 | 29.1 |  | 2 | 16,086 | 33,870 | 86,787 | 35.9 | 32.33 |
|  | 3 | 3,779 | 8,667 | 17,450 | 30.78 | 28.45 |  | 3 | 5,909 | 12,130 | 31,312 | 35.54 | 31.94 |
|  | 4 | 1,790 | 4,231 | 8,345 | 30.6 | 28.32 |  | 4 | 2,864 | 5,967 | 15,374 | 35.35 | 31.78 |
|  | 5 | 8,282 | 19,170 | 38,442 | 31.43 | 29.17 |  | 5 | 13,162 | 27,723 | 71,148 | 35.87 | 32.3 |
|  | 6 | 1,584 | 3,658 | 7,323 | 32.58 | 30.33 |  | 6 | 2,472 | 5,237 | 13,280 | 36.98 | 33.41 |
