# Supplementary material for: Climate change-driven compound dust storm and heatwave extremes in Central Asia under future CMIP6 scenarios
Source: PLoS One. 2026 Nov 4;21(11):e0359347. doi: 10.1371/journal.pone.0359347 (PMC13734455; doi:10.1371/journal.pone.0359347)
Supplement: Supplementary file 6 [file pone.0359347.s006.docx]

|  | **Table S6.** The Mann-Kendall trend analysis for heatwave-related indices during historical (1992-2021), near-future (2022-2051), and far-future (2071-2100) periods under two different climate scenarios, in different clusters across CA. For Mann-Kendall significance testing, p-values were evaluated at each location, and we report the proportion of locations within each cluster where p-value < 0.05, indicating the spatial consistency of statistically significant trends. | | | | | | | | | | | | | |
| --- | --- | --- | --- | --- | --- | --- | --- | --- | --- | --- | --- | --- | --- | --- |
|  |  | ***Historical Period (1992-2021)*** | | | | |  |  | ***Historical Period (1992-2021)*** | | | | | |
| **SPSS 2-4.5** | **Cluster** | **HWD** | **HWA** | **HWF** | **HWN** | **HWM** | **SPSS 5-8.5** | **Cluster** | **HWD** | **HWA** | **HWF** | **HWN** | **HWM** |  |
|  | *Cluster 1* | -0.026 | 0.009 | 0.029 | -0.026 | 0.004 |  | *Cluster 1* | -0.026 | 0.009 | 0.029 | -0.026 | 0.004 |  |
|  | *% (p-value <0.05)* | 4.26 | 8.51 | 4.26 | 0.00 | 9.57 |  | *% (p-value <0.05)* | 4.26 | 8.51 | 4.26 | 0.00 | 9.57 |  |
|  | *Cluster 2* | 0.029 | 0.198 | 0.546 | -0.006 | 0.075 |  | *Cluster 2* | 0.029 | 0.198 | 0.546 | -0.006 | 0.075 |  |
|  | *% (p-value <0.05)* | 90.91 | 2.67 | 99.47 | 67.91 | 0.00 |  | *% (p-value <0.05)* | 90.91 | 2.67 | 99.47 | 67.91 | 0.00 |  |
|  | *Cluster 3* | 0.111 | 0.104 | 0.613 | 0.038 | 0.101 |  | *Cluster 3* | 0.111 | 0.104 | 0.613 | 0.038 | 0.101 |  |
|  | *% (p-value <0.05)* | 48.53 | 22.06 | 86.76 | 85.29 | 5.88 |  | *% (p-value <0.05)* | 48.53 | 22.06 | 86.76 | 85.29 | 5.88 |  |
|  | *Cluster 4* | 0.158 | 0.111 | 0.864 | 0.023 | 0.171 |  | *Cluster 4* | 0.158 | 0.111 | 0.864 | 0.023 | 0.171 |  |
|  | *% (p-value <0.05)* | 40.63 | 37.50 | 100.00 | 100.00 | 0.00 |  | *% (p-value <0.05)* | 40.63 | 37.50 | 100.00 | 100.00 | 0.00 |  |
|  | *Cluster 5* | 0.046 | 0.135 | 0.442 | 0.001 | 0.076 |  | *Cluster 5* | 0.046 | 0.135 | 0.442 | 0.001 | 0.076 |  |
|  | *% (p-value <0.05)* | 76.47 | 9.80 | 84.97 | 69.28 | 0.65 |  | *% (p-value <0.05)* | 76.47 | 9.80 | 84.97 | 69.28 | 0.65 |  |
|  | *Cluster 6* | 0.061 | 0.281 | 0.960 | -0.008 | 0.136 |  | *Cluster 6* | 0.061 | 0.281 | 0.960 | -0.008 | 0.136 |  |
|  | *% (p-value <0.05)* | 79.31 | 13.79 | 100.00 | 96.55 | 0.00 |  | *% (p-value <0.05)* | 79.31 | 13.79 | 100.00 | 96.55 | 0.00 |  |
|  |  | ***Near-Future (2022-2051)*** | | | | |  |  | ***Near-Future (2022-2051)*** | | | | | |
|  | *Cluster 1* | 0.137 | 0.159 | 0.658 | 0.144 | 0.067 |  | *Cluster 1* | 0.130 | 0.231 | 0.980 | 0.183 | 0.134 |  |
|  | *% (p-value <0.05)* | 98.94 | 100 | 100 | 0 | 100 |  | *% (p-value <0.05)* | 41.49 | 10.64 | 100 | 8.51 | 100 |  |
|  | *Cluster 2* | 0.141 | 0.151 | 0.650 | 0.140 | 0.057 |  | *Cluster 2* | 0.135 | 0.216 | 1.000 | 0.185 | 0.116 |  |
|  | *% (p-value <0.05)* | 98.22 | 100 | 100 | 0 | 100 |  | *% (p-value <0.05)* | 25.44 | 7.10 | 100 | 5.92 | 100 |  |
|  | *Cluster 3* | 0.136 | 0.158 | 0.628 | 0.135 | 0.060 |  | *Cluster 3* | 0.148 | 0.229 | 0.980 | 0.178 | 0.131 |  |
|  | *% (p-value <0.05)* | 94.03 | 98.51 | 100 | 0 | 100 |  | *% (p-value <0.05)* | 50.75 | 37.31 | 100 | 16.42 | 100 |  |
|  | *Cluster 4* | 0.137 | 0.157 | 0.649 | 0.140 | 0.064 |  | *Cluster 4* | 0.135 | 0.225 | 0.990 | 0.184 | 0.125 |  |
|  | *% (p-value <0.05)* | 93.75 | 100 | 100 | 0 | 100 |  | *% (p-value <0.05)* | 34.38 | 9.38 | 100 | 3.13 | 100 |  |
|  | *Cluster 5* | 0.142 | 0.145 | 0.655 | 0.142 | 0.052 |  | *Cluster 5* | 0.136 | 0.217 | 1.006 | 0.186 | 0.117 |  |
|  | *% (p-value <0.05)* | 97.10 | 100 | 100 | 0 | 100 |  | *% (p-value <0.05)* | 23.91 | 2.90 | 100 | 2.90 | 100 |  |
|  | *Cluster 6* | 0.139 | 0.143 | 0.639 | 0.138 | 0.051 |  | *Cluster 6* | 0.147 | 0.219 | 0.995 | 0.183 | 0.117 |  |
|  | *% (p-value <0.05)* | 100 | 100 | 100 | 0 | 100 |  | *% (p-value <0.05)* | 43.48 | 8.70 | 100 | 4.35 | 100 |  |
|  |  | ***Far-Future (2071-2100)*** | | | | |  |  | ***Far-Future (2071-2100)*** | | | | | |
|  | *Cluster 1* | 0.048 | -0.026 | 0.204 | 0.020 | -0.024 |  | *Cluster 1* | 0.382 | 0.140 | 1.459 | 0.235 | -0.063 |  |
|  | *% (p-value <0.05)* | 0 | 0 | 2.13 | 0 | 0 |  | *% (p-value <0.05)* | 8.51 | 100 | 100 | 0 | 100 |  |
|  | *Cluster 2* | 0.051 | -0.023 | 0.203 | 0.019 | -0.017 |  | *Cluster 2* | 0.397 | 0.143 | 1.464 | 0.234 | -0.053 |  |
|  | *% (p-value <0.05)* | 0 | 0 | 1.18 | 0 | 0 |  | *% (p-value <0.05)* | 5.92 | 100 | 100 | 1.18 | 100 |  |
|  | *Cluster 3* | 0.053 | -0.013 | 0.211 | 0.022 | -0.018 |  | *Cluster 3* | 0.347 | 0.141 | 1.433 | 0.240 | -0.066 |  |
|  | *% (p-value <0.05)* | 0 | 1.49 | 0 | 0 | 0 |  | *% (p-value <0.05)* | 7.46 | 98.51 | 100 | 0 | 100 |  |
|  | *Cluster 4* | 0.049 | -0.015 | 0.210 | 0.021 | -0.020 |  | *Cluster 4* | 0.386 | 0.137 | 1.465 | 0.235 | -0.062 |  |
|  | *% (p-value <0.05)* | 0 | 3.13 | 0 | 0 | 0 |  | *% (p-value <0.05)* | 0 | 100 | 100 | 0 | 100 |  |
|  | *Cluster 5* | 0.053 | -0.026 | 0.211 | 0.020 | -0.017 |  | *Cluster 5* | 0.419 | 0.141 | 1.475 | 0.232 | -0.051 |  |
|  | *% (p-value <0.05)* | 0.73 | 0 | 5.11 | 0.73 | 0 |  | *% (p-value <0.05)* | 5.07 | 100 | 100 | 0 | 100 |  |
|  | *Cluster 6* | 0.050 | -0.021 | 0.206 | 0.020 | -0.017 |  | *Cluster 6* | 0.395 | 0.129 | 1.421 | 0.223 | -0.059 |  |
|  | *% (p-value <0.05)* | 0 | 0 | 0 | 0 | 0 |  | *% (p-value <0.05)* | 4.35 | 100 | 100 | 0 | 100 |  |
