# Supplementary material for: Climate change-driven compound dust storm and heatwave extremes in Central Asia under future CMIP6 scenarios
Source: PLoS One. 2026 Nov 4;21(11):e0359347. doi: 10.1371/journal.pone.0359347 (PMC13734455; doi:10.1371/journal.pone.0359347)
Supplement: Supplementary file 7 [file pone.0359347.s007.docx]

| **Table S7.** Theil-Sen slope analysis of dust level changes across CA, divided into six clusters, under historical and future climate scenarios (SSP2–4.5 and SSP5–8.5) during warm periods between 1992 and 2100. For Mann-Kendall significance testing, p-values were evaluated at each location, and we report the proportion of locations within each cluster where p-value < 0.05, indicating the spatial consistency of statistically significant trends. | | | | | | |
| --- | --- | --- | --- | --- | --- | --- |
| ***Historical Period (1992-2021)*** | | | | | | |
|  | **Cluster 1** | **Cluster 2** | **Cluster 3** | **Cluster 4** | **Cluster 5** | **Cluster 6** |
| ***Maximum*** | 5.61 | 2.62 | 0.60 | 9.19 | 9.25 | 2.09 |
| ***Minimum*** | -2.27 | -0.43 | -1.26 | -3.36 | 0.75 | -2.47 |
| ***Average*** | 1.53 | 0.58 | -0.16 | 4.17 | 5.65 | 0.01 |
| ***STD*** | 1.28 | 0.71 | 0.27 | 3.97 | 1.66 | 1.06 |
| ***% (p-value <0.05)*** | 34.34 | 58.9 | 42.86 | 33.33 | 60 | 56.41 |
| ***Near-Future SSP2-4.5 (2022-5051)*** | | | | | | |
|  | **Cluster 1** | **Cluster 2** | **Cluster 3** | **Cluster 4** | **Cluster 5** | **Cluster 6** |
| ***Maximum*** | 15.91 | 15.91 | 15.91 | 15.29 | 15.30 | 2.98 |
| ***Minimum*** | -15.91 | -15.91 | -15.91 | -15.91 | -15.91 | -15.91 |
| ***Average*** | 0.07 | -1.77 | 3.18 | -2.37 | -5.71 | -1.93 |
| ***STD*** | 7.01 | 7.19 | 7.78 | 8.05 | 6.70 | 5.15 |
| ***% (p-value <0.05)*** | 72.73 | 76.26 | 70.13 | 76.92 | 73.14 | 67.5 |
| ***Far-Future SSP2-4.5 (2071-2100)*** | | | | | | |
|  | **Cluster 1** | **Cluster 2** | **Cluster 3** | **Cluster 4** | **Cluster 5** | **Cluster 6** |
| ***Maximum*** | 15.03 | 15.53 | 14.61 | 16.45 | 13.95 | 9.89 |
| ***Minimum*** | -16.25 | -13.75 | -15.32 | -15.71 | -16.47 | -13.54 |
| ***Average*** | 0.32 | -0.59 | -0.84 | -0.90 | -1.38 | -1.37 |
| ***STD*** | 5.54 | 6.04 | 5.59 | 6.21 | 6.28 | 5.82 |
| ***% (p-value <0.05)*** | 78.79 | 72.6 | 72.73 | 71.79 | 73.14 | 70 |
| ***Near-Future SSP5-8.5 (2022-5051)*** | | | | | | |
|  | **Cluster 1** | **Cluster 2** | **Cluster 3** | **Cluster 4** | **Cluster 5** | **Cluster 6** |
| ***Maximum*** | 15.91 | 15.91 | 15.91 | 5.32 | 15.91 | 15.80 |
| ***Minimum*** | -15.91 | -15.91 | -15.91 | -15.91 | -15.91 | -15.91 |
| ***Average*** | -4.65 | -1.24 | -3.67 | -5.53 | -8.05 | -5.14 |
| ***STD*** | 6.17 | 6.24 | 7.39 | 6.25 | 6.17 | 7.93 |
| ***% (p-value <0.05)*** | 71.72 | 77.98 | 75.32 | 74.36 | 72 | 70 |
| ***Far-Future SSP5-8.5 (2071-2100)*** | | | | | | |
|  | **Cluster 1** | **Cluster 2** | **Cluster 3** | **Cluster 4** | **Cluster 5** | **Cluster 6** |
| ***Maximum*** | 16.17 | 11.95 | 11.38 | 16.47 | 16.47 | 8.49 |
| ***Minimum*** | -16.47 | -14.23 | -16.36 | -16.10 | -16.47 | -12.09 |
| ***Average*** | -3.26 | -2.30 | -4.17 | -1.81 | -2.62 | -3.65 |
| ***STD*** | 6.39 | 5.13 | 4.97 | 6.72 | 6.78 | 4.48 |
| ***% (p-value <0.05)*** | 82.83 | 80.73 | 74.03 | 76.92 | 73.71 | 77.5 |
