# Supplementary material for: Climate change-driven compound dust storm and heatwave extremes in Central Asia under future CMIP6 scenarios
Source: PLoS One. 2026 Nov 4;21(11):e0359347. doi: 10.1371/journal.pone.0359347 (PMC13734455; doi:10.1371/journal.pone.0359347)
Supplement: Supplementary file 8 [file pone.0359347.s008.docx]

| **Table S8.** Dust intensity frequencies and percentage changes across CA, divided into six clusters, under historical and future climate scenarios (SSP2–4.5 and SSP5–8.5) during warm periods between 1992 and 2100. | | | | | | | | |
| --- | --- | --- | --- | --- | --- | --- | --- | --- |
| **Cluster** | **Low Intensity** | **Moderate Intensity** | **High Intensity** | **Very High Intensity** | **Low Intensity %** | **Moderate Intensity %** | **High Intensity %** | **Very High Intensity %** |
|  | ***Historical Period (1992-2021)*** | | | | | | | |
| **1** | 5,196 | 16,576 | 2,221 | 342 | 100 | 100 | 100 | 100 |
| **2** | 6,231 | 6,591 | 875 | 253 | 100 | 100 | 100 | 100 |
| **3** | 11,402 | 3,282 | 40 | 1 | 100 | 100 | 100 | 100 |
| **4** | 0 | 1,703 | 2,027 | 4,640 | 0 | 100 | 100 | 100 |
| **5** | 1,047 | 20,339 | 9,626 | 4,793 | 100 | 100 | 100 | 100 |
| **6** | 1,506 | 1,681 | 182 | 41 | 100 | 100 | 100 | 100 |
|  | ***Near-Future SSP2***–***4.5 (2022-5051)*** | | | | | | | |
| **1** | 924 | 8,410 | 62 | 0 | 17.8 | 50.7 | 2.8 | 0.0 |
| **2** | 3,012 | 2,265 | 175 | 0 | 48.3 | 34.4 | 20.0 | 0.0 |
| **3** | 4,567 | 189 | 0 | 0 | 40.1 | 5.8 | 0.0 | 0.0 |
| **4** | 0 | 0 | 720 | 2,644 | 0.0 | 0.0 | 35.5 | 57.0 |
| **5** | 0 | 7,425 | 5,174 | 1,321 | 0.0 | 36.5 | 53.8 | 27.6 |
| **6** | 401 | 295 | 0 | 0 | 26.6 | 17.5 | 0.0 | 0.0 |
|  | ***Near-Future SSP5***–***8.5 (2022-2051)*** | | | | | | | |
| **1** | 1,130 | 7,629 | 57 | 0 | 21.7 | 46.0 | 2.6 | 0.0 |
| **2** | 2,774 | 1,851 | 247 | 0 | 44.5 | 28.1 | 28.2 | 0.0 |
| **3** | 6,320 | 408 | 0 | 0 | 55.4 | 12.4 | 0.0 | 0.0 |
| **4** | 0 | 0 | 411 | 2,489 | 0.0 | 0.0 | 20.3 | 53.6 |
| **5** | 11 | 6,612 | 5,045 | 1,556 | 1.1 | 32.5 | 52.4 | 32.5 |
| **6** | 402 | 758 | 0 | 0 | 26.7 | 45.1 | 0.0 | 0.0 |
|  | ***Far-Future SSP2***–***4.5 (2071-2100)*** | | | | | | | |
| **1** | 1,970 | 17,315 | 183 | 0 | 37.9 | 104.5 | 8.2 | 0.0 |
| **2** | 5,641 | 5,005 | 514 | 0 | 90.5 | 75.9 | 58.7 | 0.0 |
| **3** | 11,366 | 414 | 0 | 0 | 99.7 | 12.6 | 0.0 | 0.0 |
| **4** | 0 | 0 | 715 | 5,981 | 0.0 | 0.0 | 35.3 | 128.9 |
| **5** | 89 | 15,798 | 10,678 | 2,079 | 8.5 | 77.7 | 110.9 | 43.4 |
| **6** | 1,084 | 1,644 | 0 | 0 | 72.0 | 97.8 | 0.0 | 0.0 |
|  | ***Far-Future SSP5***–***8.5 (2071-2100)*** | | | | | | | |
| **1** | 2,197 | 17,036 | 219 | 16 | 42.3 | 102.8 | 9.9 | 4.7 |
| **2** | 5,552 | 4,928 | 680 | 0 | 89.1 | 74.8 | 77.7 | 0.0 |
| **3** | 11,129 | 651 | 0 | 0 | 97.6 | 19.8 | 0.0 | 0.0 |
| **4** | 0 | 0 | 870 | 5,826 | 0.0 | 0.0 | 42.9 | 125.6 |
| **5** | 41 | 16,000 | 10,273 | 2,206 | 3.9 | 78.7 | 106.7 | 46.0 |
| **6** | 1,200 | 1,528 | 0 | 0 | 79.7 | 90.9 | 0.0 | 0.0 |
