# Supplementary material for: Climate change-driven compound dust storm and heatwave extremes in Central Asia under future CMIP6 scenarios
Source: PLoS One. 2026 Nov 4;21(11):e0359347. doi: 10.1371/journal.pone.0359347 (PMC13734455; doi:10.1371/journal.pone.0359347)
Supplement: Supplementary file 9 [file pone.0359347.s009.docx]

| **Table S9.** The range of positive and negative correlations, including observed maximum and minimum correlations, between dust levels and heatwave indices (HWM, HWA, HWN, HWD, HWF) for six clusters across historical and future scenarios (SSP2–4.5 and SSP5–8.5) in CA between 1992 and 2100. | | | | | | | | | | |
| --- | --- | --- | --- | --- | --- | --- | --- | --- | --- | --- |
| **Historical period (1992-2021)** | | | | | | | | | | |
| ***Cluster*** | **HWM_Min** | **HWM_Max** | **HWA_Min** | **HWA_Max** | **HWN_Min** | **HWN_Max** | **HWD_Min** | **HWD_Max** | **HWF_Min** | **HWF_Max** |
| **1** | -0.29 | 0.45 | -0.27 | 0.47 | -0.24 | 0.62 | -0.23 | 0.54 | -0.18 | 0.64 |
| **2** | -0.31 | 0.37 | -0.27 | 0.38 | 0 | 0.45 | -0.34 | 0.38 | -0.04 | 0.44 |
| **3** | -0.26 | 0.41 | -0.27 | 0.36 | -0.33 | 0.18 | -0.35 | 0.24 | -0.33 | 0.12 |
| **4** | -0.3 | 0.39 | -0.3 | 0.43 | -0.29 | 0.63 | -0.2 | 0.56 | -0.23 | 0.63 |
| **5** | -0.11 | 0.55 | -0.04 | 0.55 | -0.07 | 0.64 | 0.01 | 0.64 | -0.07 | 0.67 |
| **6** | -0.13 | 0.2 | -0.12 | 0.23 | -0.12 | 0.49 | -0.32 | 0.34 | -0.19 | 0.46 |
| **Near-Future SSP2**–**4.5 (2022-2051)** | | | | | | | | | | |
| ***Cluster*** | **HWM_Min** | **HWM_Max** | **HWA_Min** | **HWA_Max** | **HWN_Min** | **HWN_Max** | **HWD_Min** | **HWD_Max** | **HWF_Min** | **HWF_Max** |
| **1** | -0.69 | 0.6 | -0.89 | 0.92 | -0.66 | 0.65 | -0.88 | 0.81 | -0.77 | 0.77 |
| **2** | -0.73 | 0.6 | -0.92 | 0.84 | -0.65 | 0.62 | -0.78 | 0.84 | -0.75 | 0.78 |
| **3** | -0.55 | 0.74 | -0.77 | 0.85 | -0.67 | 0.68 | -0.83 | 0.83 | -0.77 | 0.78 |
| **4** | -0.71 | 0.61 | -0.83 | 0.82 | -0.66 | 0.6 | -0.8 | 0.84 | -0.74 | 0.75 |
| **5** | -0.69 | 0.62 | -0.89 | 0.89 | -0.68 | 0.68 | -0.84 | 0.85 | -0.8 | 0.79 |
| **6** | -0.69 | 0.63 | -0.86 | 0.77 | -0.66 | 0.54 | -0.79 | 0.76 | -0.76 | 0.65 |
| **Near-Future SSP5**–**8.5 (2022-2051)** | | | | | | | | | | |
| ***Cluster*** | **HWM_Min** | **HWM_Max** | **HWA_Min** | **HWA_Max** | **HWN_Min** | **HWN_Max** | **HWD_Min** | **HWD_Max** | **HWF_Min** | **HWF_Max** |
| **1** | -0.71 | 0.55 | -0.71 | 0.57 | -0.67 | 0.62 | -0.43 | 0.33 | -0.6 | 0.56 |
| **2** | -0.6 | 0.49 | -0.54 | 0.51 | -0.66 | 0.66 | -0.43 | 0.33 | -0.58 | 0.56 |
| **3** | -0.77 | 0.61 | -0.76 | 0.69 | -0.64 | 0.62 | -0.45 | 0.3 | -0.59 | 0.54 |
| **4** | -0.62 | 0.43 | -0.64 | 0.4 | -0.64 | 0.2 | -0.41 | 0.22 | -0.59 | 0.23 |
| **5** | -0.71 | 0.59 | -0.74 | 0.47 | -0.68 | 0.61 | -0.46 | 0.38 | -0.63 | 0.61 |
| **6** | -0.57 | 0.33 | -0.5 | 0.4 | -0.65 | 0.6 | -0.38 | 0.4 | -0.59 | 0.58 |
| **Far-Future SSP2**–**4.5 (2071-2100)** | | | | | | | | | | |
| ***Cluster*** | **HWM_Min** | **HWM_Max** | **HWA_Min** | **HWA_Max** | **HWN_Min** | **HWN_Max** | **HWD_Min** | **HWD_Max** | **HWF_Min** | **HWF_Max** |
| **1** | -0.29 | 0.32 | -0.3 | 0.34 | -0.42 | 0.45 | -0.42 | 0.53 | -0.45 | 0.46 |
| **2** | -0.31 | 0.23 | -0.34 | 0.32 | -0.37 | 0.45 | -0.5 | 0.48 | -0.44 | 0.39 |
| **3** | -0.29 | 0.25 | -0.28 | 0.29 | -0.44 | 0.44 | -0.27 | 0.5 | -0.4 | 0.39 |
| **4** | -0.29 | 0.27 | -0.4 | 0.3 | -0.44 | 0.38 | -0.6 | 0.21 | -0.41 | 0.35 |
| **5** | -0.28 | 0.33 | -0.28 | 0.4 | -0.42 | 0.45 | -0.4 | 0.42 | -0.41 | 0.4 |
| **6** | -0.21 | 0.19 | -0.24 | 0.27 | -0.38 | 0.43 | -0.31 | 0.25 | -0.37 | 0.37 |
| **Far-Future SSP5**–**8.5 (2071-2100)** | | | | | | | | | | |
| ***Cluster*** | **HWM_Min** | **HWM_Max** | **HWA_Min** | **HWA_Max** | **HWN_Min** | **HWN_Max** | **HWD_Min** | **HWD_Max** | **HWF_Min** | **HWF_Max** |
| **1** | -0.28 | 0.3 | -0.59 | 0.44 | -0.85 | 0.86 | -0.65 | 0.57 | -0.88 | 0.88 |
| **2** | -0.17 | 0.24 | -0.43 | 0.38 | -0.77 | 0.83 | -0.58 | 0.6 | -0.83 | 0.88 |
| **3** | -0.34 | 0.24 | -0.43 | 0.42 | -0.88 | 0.78 | -0.61 | 0.57 | -0.89 | 0.84 |
| **4** | -0.19 | 0.42 | -0.38 | 0.4 | -0.83 | 0.83 | -0.6 | 0.55 | -0.82 | 0.83 |
| **5** | -0.41 | 0.4 | -0.54 | 0.41 | -0.84 | 0.87 | -0.64 | 0.67 | -0.88 | 0.9 |
| **6** | -0.16 | 0.19 | -0.32 | 0.45 | -0.81 | 0.75 | -0.61 | 0.55 | -0.82 | 0.77 |
