# Supplementary material for: Climate change-driven compound dust storm and heatwave extremes in Central Asia under future CMIP6 scenarios
Source: PLoS One. 2026 Nov 4;21(11):e0359347. doi: 10.1371/journal.pone.0359347 (PMC13734455; doi:10.1371/journal.pone.0359347)
Supplement: Supplementary file 10 [file pone.0359347.s010.docx]

| **Table S10.** Strongest FDR-supported dust–heatwave associations and selected SSP5–8.5 retained associations across CA. | | | | |
| --- | --- | --- | --- | --- |
| **Period** | **Scenario** | **Cluster** | **Index** | **FDR-significant locations (%)** |
| Historical | Historical | 6 | HWF | 68.0 |
| Near-Future | SSP2–4.5 | 1 | HWA | 61.1 |
| Near-Future | SSP2–4.5 | 5 | HWA | 58.4 |
| Historical | Historical | 5 | HWF | 58.0 |
| Historical | Historical | 1 | HWF | 53.8 |
| Near-Future | SSP2–4.5 | 2 | HWA | 51.6 |
| Near-Future | SSP2–4.5 | 2 | HWM | 48.4 |
| Near-Future | SSP2–4.5 | 1 | HWM | 45.8 |
| Far-Future | SSP5–8.5 | 3 | HWN | 19.34 |
| Far-Future | SSP5–8.5 | 3 | HWF | 14.36 |
| **Note:** The first eight rows summarize the strongest FDR-supported associations overall. The final two rows are included to show retained FDR-significant associations under SSP5–8.5 in Cluster 3, corresponding to northeastern Kazakhstan. These SSP5–8.5 associations are weaker than the strongest historical and SSP2–4.5 signals but remain relevant for interpreting localized dust–heatwave co-variability. | | | | |
